# Measuring ROS and redox markers in plant cells†

**DOI:** 10.1039/d1cb00071c

**Published:** 2021-06-29

**Authors:** Salma Akter, Mohammad Shahneawz Khan, Edward N. Smith, Emily Flashman

**Affiliations:** Department of Chemistry, University of Oxford Oxford UK emily.flashman@chem.ox.ac.uk; Faculty of Biological Sciences, University of Dhaka Dhaka 1000 Bangladesh; Department of Plant Sciences, University of Oxford UK

## Abstract

Reactive oxygen species (ROS) are produced throughout plant cells as a by-product of electron transfer processes. While highly oxidative and potentially damaging to a range of biomolecules, there exists a suite of ROS-scavenging antioxidant strategies that maintain a redox equilibrium. This balance can be disrupted in the event of cellular stress leading to increased ROS levels, which can act as a useful stress signal but, in excess, can result in cell damage and death. As crop plants become exposed to greater degrees of multiple stresses due to climate change, efforts are ongoing to engineer plants with greater stress tolerance. It is therefore important to understand the pathways underpinning ROS-mediated signalling and damage, both through measuring ROS themselves and other indicators of redox imbalance. The highly reactive and transient nature of ROS makes this challenging to achieve, particularly in a way that is specific to individual ROS species. In this review, we describe the range of chemical and biological tools and techniques currently available for ROS and redox marker measurement in plant cells and tissues. We discuss the limitations inherent in current methodology and opportunities for advancement.

## Abstract 2

Reactive oxygen species and redox markers in plants are measured using a range of chemical probes and biosensors.

## Graphical abstract

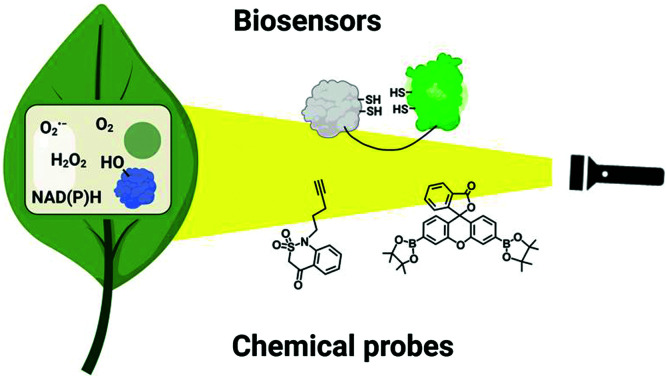

## 1. Introduction

The evolution of aerobic life 2.2 billion years ago corresponds with the ability of cells to exploit the availability of oxygen and its electron accepting ability to generate energy through oxidative phosphorylation. Through this process, NADH oxidation and oxygen reduction to water maintain a proton gradient across the mitochondrial membrane, which is the driving force for ATP synthesis. The efficiency of this process relies on tight coupling of electron transport in the mitochondrial membrane and sufficient oxygen availability to accept the electrons generated by oxidation of NADH in the electron transport chain. Any imbalance in the ratio of electrons to oxygen, *e.g.* through insufficient oxygen availability, can cause incomplete reduction of oxygen and the formation of the reactive oxygen species (ROS) superoxide (O_2_˙^−^). Superoxide formation can also occur at other locations in the cell where electrons and oxygen are prone to meet including photosynthetic electron transport chains, as well as specifically *via* NADPH oxidases (*e.g.* respiratory burst oxidase homolog, RBOH, enzymes in plants) which catalyse formation of superoxide alongside NADP^+^. The accumulation and subsequent reactions of this short-lived, readily oxidising ROS therefore generate an oxidative environment in the local region, impacting its ‘redox status’.

**Fig. 1:**
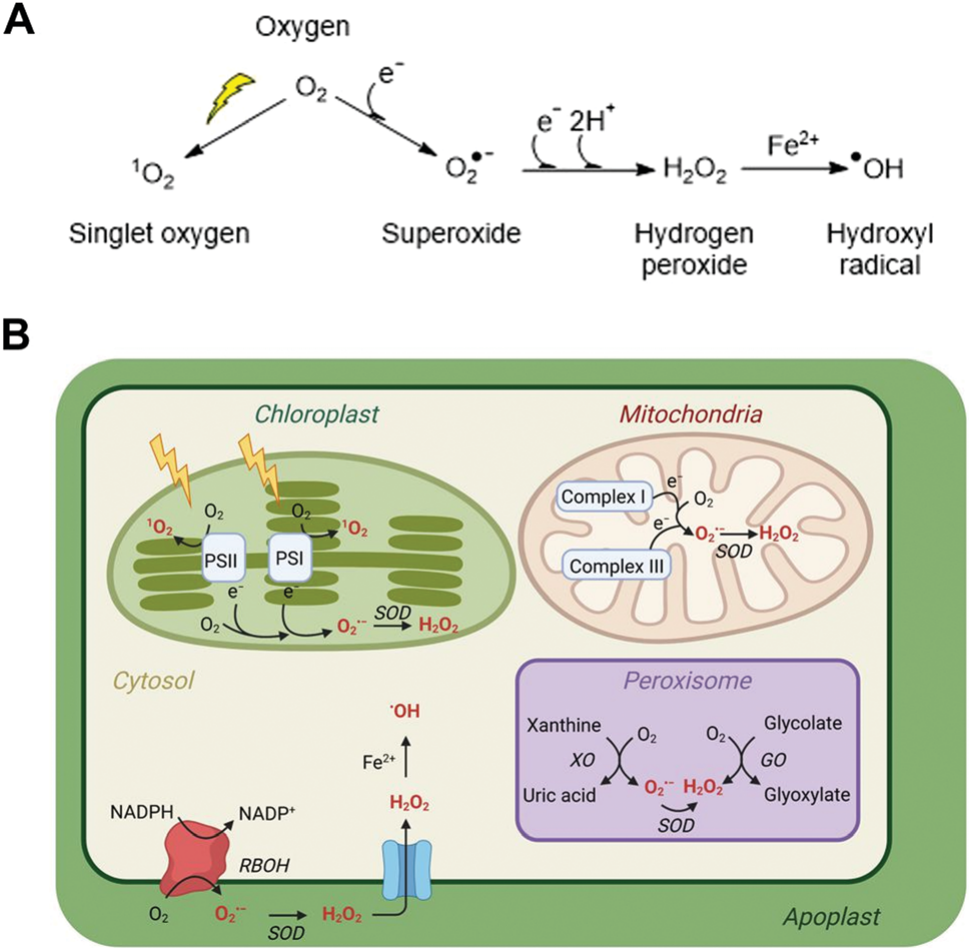
ROS in plant cells. (A) Common ROS production from atmospheric oxygen; (B) primary ROS production sites in a plant cell (for detailed production and scavenging sites see Waszczak C *et al.*^8^); PSI/II, photosystem I/II; SOD, superoxide dismutase; XO, xanthine oxidase; GO, glycolate oxidase; RBOH, respiratory burst oxidase homolog.

Superoxide can be converted into other ROS (Fig. 1A) and the impact of the altered redox status in a cell or subcellular region depends very much on the nature, location, intensity and duration of the species formed. ROS have the potential to be damaging to a range of important biomolecules in the cell, including proteins, lipids and nucleic acids. However, there are multiple mechanisms in place to ‘mop up’ excessive ROS species in the form of both small molecule buffers (*e.g.* glutathione, ascorbate) and enzymes (*e.g.* superoxide dismutase, catalase). This balance of ROS formation and ROS scavengers enables cells to survive inevitable ROS production without excessive damage.

Nevertheless, this balance can become disrupted in certain, usually stressful, conditions. In plants, such conditions include pathogen attack, heat, drought and flooding.^1^ An increase in ROS levels beyond the ability of the cell to buffer the oxidative activity will increase the oxidative status of the cell. The effects of this can, to a degree, be beneficial, acting as a signal for stress responses as well as development and organ growth.^2,3^ ROS signalling is indeed intrinsically linked to hormone signalling including with abscisic acid and drought stress, ethylene-induced stomata closure and with salicylic acid and pathogen defence.^4^ However, if this balance is tipped too far, the overall effects can be detrimental, ultimately resulting in cell death. In the case of plants, that means the stress-induced ROS-mediated damage can have an impact on crop health and survival.^5^

As part of efforts to generate crops that are resilient to emerging climate-related hazards, it is important to understand ROS flux and the mechanisms by which ROS signal for growth or stress responses. It is therefore necessary to correlate the presence of different ROS species and the associated outcome. But this is extremely challenging when ROS are by their very nature so reactive and therefore short-lived. Genuine detection and quantification of individual ROS species in a temporal and spatially relevant manner is, to date, an elusive goal. Nevertheless, efforts are ongoing to develop meaningful sensors of redox modulation.

In this review, we provide an overview of the state-of-the-art with respect to the methods and tools used to detect and quantify both ROS directly and other fluctuating redox markers in plant cells, including the resultant oxidative modifications. We identify their limitations and consider what is needed to advance the tools and techniques in this field. Many of the methods used in plants are also used in studies of mammalian redox regulation, however, not all tools used in mammalian studies can be applied to plants due to certain inherent limitations such as cell permeability and intrinsic fluorescent components in plant cells.

Alongside this overview, readers are encouraged to refer to the ESI† accompanying this review, which comprises tables with additional details for the probes and methods discussed in the main text. These include whether they are qualitative or quantitative, their ability to provide temporal or spatial resolution and additional specific examples of biological questions they have been used to address. We hope that this review will inspire the Chemical Biology community to tackle the remaining challenges in effective measurement of ROS and redox markers in plants.

## 2. Production of reactive oxygen species in plant cells

ROS is an umbrella term applied to a collection of small molecules that contain one or more atoms of oxygen that have acquired electrons or energy to form radicals. These species are short-lived but highly reactive and capable of oxidising a range of biomolecules. The main ROS species with impacts in cells are superoxide (O_2_˙^−^), hydrogen peroxide (H_2_O_2_), the hydroxide radical (˙OH) and singlet oxygen (^1^O_2_); these are significantly more reactive than native triplet oxygen (Fig. 1A). Nitric oxide (NO˙) and peroxynitrite (ONOO^−^) are known as reactive nitrogen species (RNS) and can also contribute to oxidative stress, although for reasons of space we do not discuss methods for their measurement in this review and refer readers elsewhere.^6^ Each type of ROS is distinct according to its chemical properties and half-life in biological systems.^7,13^

### Superoxide, O_2_˙^−^

O_2_˙^−^ is a highly reactive but short-lived ROS (*t*_1/2_ = 1–4 μs, though this is context dependent), predominantly produced in plant cells as a result of electron leakage from the electron transport chains of mitochondria (complexes I and III) and chloroplasts (photosystems I and II). This electron leakage is well managed, with antioxidant enzymes present in these organelles to ‘mop up’ the O_2_˙^−^ produced and prevent oxidative damage. In the mitochondria and chloroplasts, these comprise superoxide dismutase enzymes (SODs), which catalyse disproportionation of O_2_˙^−^ to O_2_ and H_2_O_2_. The H_2_O_2_ is then detoxified by ascorbate or glutathione peroxidases, coupled to ascorbate/glutathione reductases. O_2_˙^−^ is also produced in peroxisomes through the activity of xanthine oxidases and *via* leakage from an electron transport chain, as well as in the apoplast through the activity of RBOH enzymes.

### Hydrogen peroxide, H_2_O_2_

H_2_O_2_ is the most stable of the ROS, with a lifetime of up to 1 s, even increasing to 10 s in *Arabidopsis thaliana* (hereafter Arabidopsis) guard cells.^9^ As described above, it is produced through SOD-catalysed dismutation of O_2_˙^−^ throughout the cell. H_2_O_2_ is particularly significant in the apoplast as a result of RBOH activity at the cell membrane: H_2_O_2_ is more long-lived in the apoplast compared to other parts of the cell due to the absence of enzymes that catalyse its removal. Consequently, apoplastic H_2_O_2_ is an important signalling molecule; it is able both to re-enter the cytoplasm *via* aquaporin membrane proteins to react with cytoplasmic proteins, and it can react with cysteine residues on plasma membrane surface receptor proteins to initiate Ca^2+^ influx and consequent stress response mechanisms.^10^

### The hydroxyl radical, ˙OH

˙OH is very short-lived, with a half-life in the range of nanoseconds. It is extremely reactive and does not diffuse readily through the cell, being diffusion limited to <1 nm. It is formed through addition of an electron to H_2_O_2_, typically *via* Fenton chemistry whereby a transition metal, *e.g.* Fe^2+^, is oxidised by H_2_O_2_. Ascorbate, which is present at high concentrations in plant cells, can reduce Fe^3+^ back to Fe^2+^, enabling repeated ˙OH production. There are no specific ˙OH scavengers; it is promiscuous in its targets, but removal of transition metals through chelation can reduce its formation.

### Singlet oxygen, ^1^O_2_

^1^O_2_ is formed through the activation of O_2_ by light absorption. It increases under photo-oxidative stress, and most ^1^O_2_ in plants is produced in the chloroplasts of mesophyll cells in leaves, though it is produced elsewhere in cells as well.^11^ It is short-lived (lifetime of <10 μs in cell culture), but can diffuse >100 nm including through membranes.^11^ As such, although it reacts rapidly with a range of molecules within the chloroplast, *e.g.* beta-carotene, tocopherol, flavonoids and proteins, it can also migrate to other parts of the cell. ^1^O_2_ can initiate signalling processes which result either in programmed cell death or acclimation to high light stress.

## 3. Direct measurement of ROS species using chemical probes, biosensors and electron paramagnetic resonance

To understand the role of ROS in redox status dysregulation, cellular signalling and damage, it is necessary to measure ROS levels directly, rather than measure the downstream effects of their activity. ROS however, as described above, are short-lived and therefore inherently difficult to detect, particularly *in vivo*. As H_2_O_2_ and O_2_˙^−^ radicals are relatively stable, most ROS-detection methods seek to quantify these species. Electron paramagnetic resonance can directly detect ROS using spin traps and probes (Section 3.3), although most methods quantifying ROS do so through chemical probes (Section 3.1) or biosensors (Section 3.2). For additional details regarding the specific probes described, please see Table S1 (ESI†).

### 3.1 Chemical probes

Chemical probes that (in principle) preferentially react with a specific ROS form are widely used for histochemical staining or fluorescent detection of ROS. Oxidation of the probe by the ROS leads to a colorimetric change or induces fluorescence. Here, we summarise the applications of those in widespread use for plant cells and tissues, but a detailed description of chemical probes claiming their specificity to particular ROS is also enlisted in the review of Mattila *et al.*^12^

#### Superoxide

**Fig. 2:**
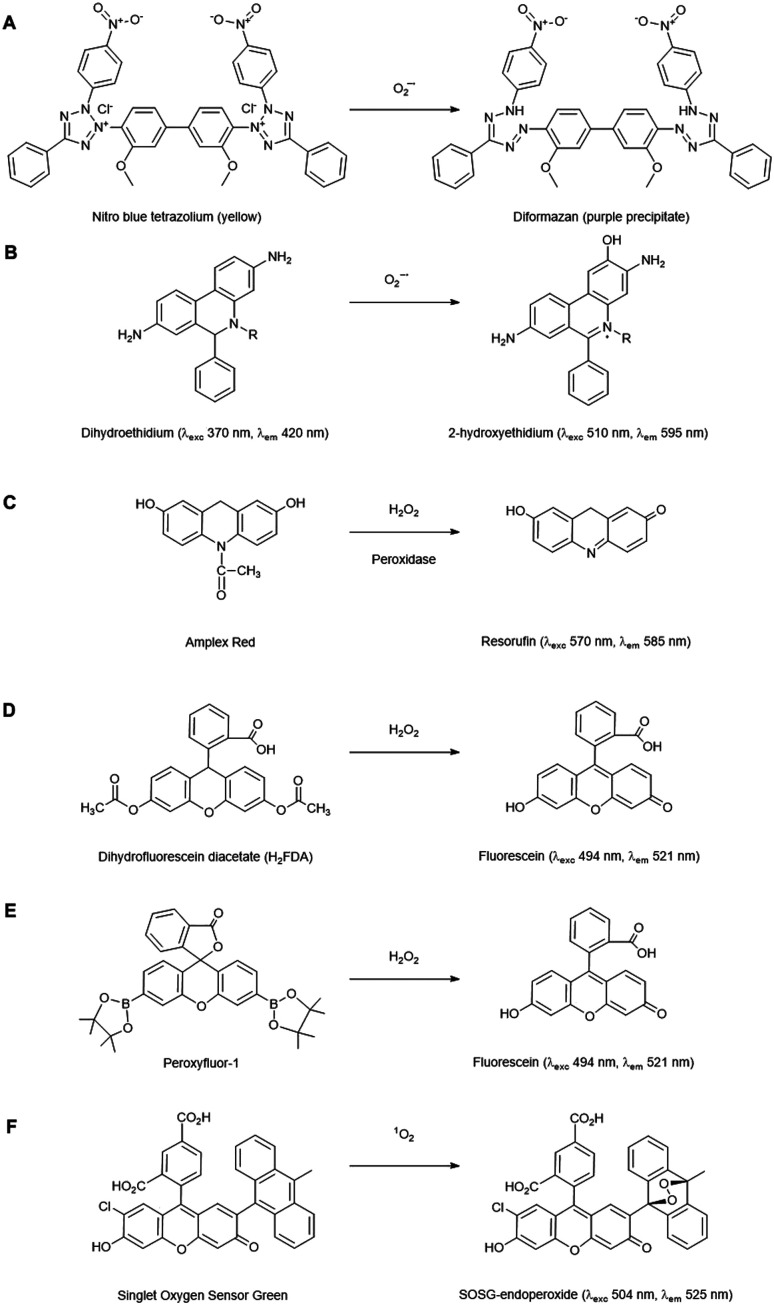
Representative ROS-probes currently used in plant cells. (A) Nitro blue tetrazolium (NBT), (B) Dihydroethidium, (C) Amplex Red, (D) Dihydrofluoroscein diacetate, (E) Peroxyfluor-1 and (F) Singlet oxygen sensor green.

A few methods claim to detect the O_2_˙^−^ radical. Tetrazolium dyes are frequently used *in situ*, *e.g.* nitro blue tetrazolium (NBT) and 3′-(1-[phenylamino-carbonyl]-3,4-tetrazolium)-bis(4-methoxy-6-nitro)benzene-sulfonic acid hydrate (XTT), both of which have been used in photosynthetic organisms for histochemical staining.^13,14^ The tetrazolium ring is opened upon four electron reduction by O_2_˙^−^ to form a diformazan product (Fig. 2A), resulting in a colour change. Although widely used to detect tissue-level O_2_˙^−^ formation, these salts are not specific for direct measurement of O_2_˙^−^ in a cellular environment as their reduction can also be attributed to ascorbate and dehydrogenase enzymes. Furthermore, oxygen availability can influence NBT reduction rates and NBT itself can promote O_2_˙^−^ formation.^15,16^

The fluorescent probe dihydroethidium (also known as hydroethidine) shows blue fluorescence in the cytosol in its reduced form, but upon four electron oxidation by O_2_˙^−^ forms 2-hydroxyethidium which intercalates with DNA, shifting its excitation and emission peaks resulting in red fluorescence (Fig. 2B). This has been used to detect O_2_˙^−^ levels in a number of plant studies, *e.g.* investigating toxicity responses in pea and rice.^17,18^ Derivatives of this dye have also been developed which localise to specific cellular regions, *e.g.* MitoSOX which has detected O_2_˙^−^ in the mitochondria of primary astrocytes of rat^19^ and, subsequently, mitochondrial isolates of *Arabidopsis* protoplasts.^20^ 2-Electron oxidation by other oxidants can form the ethidium cation, which can also bind to nuclear DNA and fluoresce red, thus competing with the O_2_˙^−^ oxidised product. As 2-hydroxyethidium is thought to be exclusively formed by the reaction with O_2_˙^−^, meaningful quantitation of this ROS requires extraction and chromatographic quantification of the specific product.^21^

#### Hydrogen peroxide

As the most stable of the ROS, a relatively high number of methods and probes exist to detect H_2_O_2_ in plants. Cerium chloride can react with extracellular H_2_O_2_ to form cerium perhydroxide precipitates, visualised through transmission electron microscopy^22^ and 3,3′-diaminobenzidine (DAB) polymerises to form a precipitate when oxidised by a peroxidase in the presence of H_2_O_2_.^23^ 3-Methyl-2-benzothiazoline hydrazone (MBTH) and 3-(dimethylamino)benzoic acid (DMAB) also react with H_2_O_2_ in a peroxidase-catalyzed reaction to form a spectrophotometrically visible product, however, their specificity is questionable *in planta*. Luminol (3-amino-phthal-hydrazide) is oxidized by peroxidase in the presence of H_2_O_2_ to yield blue luminescence, however, the signal is quenched by many cellular components.^24^

There are a number of fluorescent probes available to detect H_2_O_2_. Amplex Red (*N*-acetyl-3,7-dihydroxyphenoxazine) and its derivatives are substrates for peroxidases, such that in the presence of H_2_O_2_, the fluorescent resorufin is formed (Fig. 2C). This has been used to detect H_2_O_2_ production from a range of plant types.^25^ Nevertheless, *in vivo* use of such probes has distinct challenges: Amplex Red is not cell permeable so it can only measure extracellular H_2_O_2_, light-mediated photochemical oxidation of resorufin can occur in the presence of reducing metabolites,^26^ glutathione and NADH can induce Amplex Red oxidation^27^ and a study in leaf tissue has suggested that they can experience photo-bleaching and negatively affect PSII photochemical yield, thus their presence alone in plant cells can induce stress.^28^

The dihydrofluorescein group of dyes are widely used in plants to detect intracellular H_2_O_2_, including dihydrofluorescein diacetate (H_2_FDA), 2′,7′-dichlorodihydrofluorescein diacetate (H_2_DCFDA) and 5- (and 6-) chloromethyl-2′,7′ dichlorodihydrofluorescein diacetate (CM-H_2_DCFDA). In contrast to Amplex Red, these diacetate esters are cell permeable, allowing intracellular accumulation before reaction with H_2_O_2_ to form fluorescent products (Fig. 2D). These dyes are, however, nonspecific, reacting with a wide range of radical-based reactive species and oxidative reagents; they can also be susceptible to photooxidation and photobleaching, and can even artificially amplify ROS levels.^25,29^ Nevertheless, they have found utility as intracellular agents and have been used widely to reveal biological roles for H_2_O_2_, *e.g.* in ethylene-induced stomatal closure.^30^ Of the commonly used agents these probably have the best spatial and temporal resolution properties.

Boronate probes have been developed over recent years whereby oxidative deboronation by H_2_O_2_, hypochlorite or peroxynitrite leads to release of a reporter molecule,^31^*e.g.* Peroxyfluor-1 (PF1), a diboronate derivative of fluorescein which is oxidised in cells resulting in fluorescence (Fig. 2E). A number of derivatives have been produced, including some able to accumulate in specific sub-cellular locations. These have been used in a range of mammalian studies, but they have also been trialled in Arabidopsis cells and tissues to determine the primary source of H_2_O_2_ production in a model designed to mimic pathogen attack.^32^ Whilst these probes have greater specificity than some of their predecessors, they are still not completely specific to H_2_O_2_ detection, being readily oxidised by HOCl and ONOO^−^, so caution is still needed in their application and interpretation. Nevertheless, this type of redox-activated fluorescent probe is an interesting area for potential development as the chemical properties required for activation can be manipulated to optimise specificity towards certain ROS/RNS species or targeting to subcellular regions; the parent fluorophore can also be used to aid quantification.

#### Singlet oxygen

Singlet oxygen measurement has been achieved in plants using fluorescence-based probes such as DanePy (3-[*N*-(β-diethylaminoethyl)-*N*-dansyl]aminomethyl-2,2,5,5-tetramethyl-2,5-dihydro-1*H*-pyrrole) and Singlet Oxygen Sensor Green (SOSG). The fluorescence of DanePy is quenched by ^1^O_2_, however O_2_˙^−^, HO˙, H_2_O_2_ and lipid peroxidation products can also contribute to this fluorescence quenching. In addition, the presence of ^1^O_2_ scavengers like histidine can reduce the degree of fluorescence quenching observed.^33^ SOSG contains a fluorophore but an anthracene moiety quenches the fluorophore by photo-induced electron transfer. When the anthracene reacts with ^1^O_2_ it forms an endoperoxide, which prevents this quenching activity, resulting in green fluorescence upon light excitation (Fig. 2F).^34^ Poor penetration, UV photobleaching and high photosensitivity causing a reduced yield of photosystem II are some major drawbacks of this probe, limiting its application in photosynthetic systems.^35,36^

Overall, criticisms persist for most of the chemical probes in use for ROS measurement due to factors including their lack of chemical specificity, potential to induce ROS formation and insufficient spatial and temporal resolution.^29^ Care should therefore be taken before making firm conclusions from ROS measurements in plants using chemical probes. Detailed guidelines are presented by Noctor *et al.*^15^ where the authors emphasise the need to use several methods to cross check ROS measurements, a practise that is widely used.

The ability of some probes to react with multiple ROS can, in fact, be exploited to determine overall ROS levels in response to a range of stimuli. One such method has recently been developed for *in vivo* imaging of the systemic ROS response arising from biotic and abiotic stress in whole Arabidopsis plants in soil.^37^ H_2_DCFDA, which reacts with H_2_O_2_, HO˙, O_2_˙^−^ and ONOO^−^, was applied to Arabidopsis plants and found to penetrate cells to measure both intracellular and apoplastic ROS accumulation. Individual leaves were imaged upon exposure to a range of stresses, with a concurrent increase in ROS-induced fluorescence. Interestingly, increased fluorescence was also seen in leaves that were not subject to the stress treatment, demonstrating mobility of ROS signalling. This method was also shown to be compatible with crop plants like maize and wheat where whole-plant ROS levels were measured in response to wounding.^37^ Although crude in terms of distinguishing the species involved, this method could prove useful for quantifying and comparing redox stress in different experimental groups of plants.

Finally, an exciting area of development for quantitative ROS-sensing in plants is through the use of nanoprobes. A hybrid nano-sensing probe consisting of silicon oxide quantum dots (Si–O QDs) and silver nanoclusters (Ag NCs) has been used to measure H_2_O_2_ directly in Lettuce mitochondria *in vitro*, and *in vivo* imaging of H_2_O_2_ was also reported following wounding.^38^ The intrinsic fluorescence of the Ag NCs is quenched by H_2_O_2_ thus can act as a signal for H_2_O_2_ detection, and while the Si–O QD signal is also quenched, it gradually recovers whereas that of the Ag-NCs decreases at two different wavelengths, meaning the fluorescence ratio can be used to quantify H_2_O_2_ accurately. Unfortunately, for this nanohybrid probe, *in vivo* chloroplast fluorescence overlapped with Ag NC fluorescence, meaning a two-signal ratio measurement was not attainable for green tissue, although this approach could certainly be developed. A more successful approach has been the development of H_2_O_2_ nanosensors based on near-infrared (nIR) fluorescent single-walled carbon nanotubes (SWCNTs). The SWCNTs are functionalised, *e.g.* with a DNA aptamer that binds hemin; when this reacts with H_2_O_2_, nIR emission is quenched allowing a ratiometric measurement of H_2_O_2_*in vivo.* These have been successfully applied to selectively monitor the stress-induced production of H_2_O_2_ in a range of plants.^39–41^ This approach has a number of advantages, namely that the functionalization of the SWCNTs can be modified for specific ROS detection, they can be applied non-invasively and the arising signal can be detected remotely meaning this approach can actually be applied in the field to detect a range of stresses.^40^

### 3.2 Fluorescent biosensors

Biosensors are proteins, or other biological molecules, that detect the presence of a signal and elicit a response. The ability of some proteins to react with ROS has been exploited to engineer a range of ROS biosensors which can be expressed in plant cells (for recent review see Müller-Schüssele *et al.*^42^). Biosensors undergo a change in conformation upon reaction with ROS, which in turn elicits a fluorescent response. This can occur through direct modification of a fluorescent protein, or a conjugated system comprising a sensor protein linked to a fluorescent protein. Fluorescent biosensors offer a number of advantages over chemical probes: as they are genetically-encoded, they are non-invasive meaning the addition and cell permeation of chemical probe are not necessary. They can be temporally controlled through inducible expression and spatially controlled through targeting of the biosensors to specific organelles. These biosensors can also show specificity towards distinct ROS (typically H_2_O_2_) as well as reversibility which enables the distinct advantage of monitoring dynamic changes of redox status.^43^

The conjugated fluorescent protein (FP) probes used in redox or ROS sensing are generally based on green fluorescence protein (GFP). Its chromophore forms easily through intramolecular cyclization and oxidation of the amino acids Ser65, Tyr66 and Gly67, and it has dual excitation maxima deriving from the protonated or deprotonated state of the Tyr_66_ OH.^44^ It is important to note that oxygen is required for the formation of the GFP chromophore, and while it is not often limiting, GFP-based probes may be of limited use in severely hypoxic or anoxic environments. Many modifications have been made to the original GFP including a Ser65Thr mutation to enhance fluorescence intensity (EGFP).

Redox-sensitive GFPs (roGFP1 from wtGFP and roGFP2 from EGFP) have been engineered to incorporate a pair of redox-sensitive Cys residues on the surface of the GFP, the oxidation of which leads to disulfide bond formation and conformational changes that affect the protonation state of Tyr66. These two probes therefore offer rapid and reversible ratiometric measurement of fluorescence in response to redox changes in reducing compartments, and recombinant roGFP constructs with targeting sequences also allow plant organelle (cytosol, mitochondria, ER, peroxisomes) specific redox sensing.^45–49^ These biosensors have been used extensively in plants, for example in examining Arabidopsis responses to drought stress^50^ and saline stress^51^ (see Kostyuk *et al.*^52^ for further examples).

Although roGFP1 and roGFP2 have helped make significant progress in understanding global cellular redox status, they nevertheless lack specificity as signal arises from a change in thiol oxidation status which can have a variety of causes (including glutathione redox potential as well as ROS). Consequently, ROS-specific probes have since been developed, notably HyPer and roGFP2-Orp1, both of which are H_2_O_2_-specific chimeric constructs.

HyPer was the first genetically-encoded H_2_O_2_-specific responsive probe and comprehensive details of the HyPer probe family and their application are reviewed by Bilan and Belousov.^53^ Briefly, HyPer was created by fusion of circularly permuted yellow fluorescence protein (cpYFP) with the H_2_O_2_-responsive regulatory domain of *Escherichia coli*, OxyR.^54^ H_2_O_2_ can oxidise Cys199 of OxyR to sulfenic acid, which initiates its movement out of a hydrophobic pocket to form a disulfide bond with Cys208. This conformational remodelling of OxyR mediates a measurable spectrum change to cpYFP fluorescence that allows distinction between the oxidized and reduced state of the molecule.

Subsequent improvements have been made in the HyPer probe family regarding reaction kinetics and dynamic range, generated by incorporating mutations in the OxyR domain, *e.g.* HyPer2 (A406V),^55^ HyPer3 (A406V/H34Y)^56^ and HyPer7 (Y132F, D135N, G298S and T379P),^57^ which is a pH-stable ultrasensitive H_2_O_2_ probe. Although HyPer7 has not yet been tested in plants, other members of this family of H_2_O_2_-detecting probes has been extensively used *in vivo*, including in a range of subcellular compartments, tissues and different model plants to help understand H_2_O_2_-mediated signalling events.^53^ The pH sensitivity of the cpYFP chromophore is a major drawback of HyPer based probes,^58,59^ and local pH control or correction for pH is essential in HyPer probe based experiments, therefore, establishing HyPer7 use in plants would be highly advantageous. In the meantime, a ROS-insensitive (but still pH-sensitive) cysteine mutant, SypHer, has been generated and used as a pH control in HyPer probe experiments.^60^ This allows for identification of ROS-specific changes in fluorescence, however it also requires that this control is consistently used, including with matching SypHer variants for each variant of HyPer,^61^ bringing additional complexity to experimental and data analysis. SypHer3s (Y145F, D129G, Q197L), a brighter variant of SypHer, has recently been utilized for monitoring the pH change in zebrafish embryos,^62^ but is yet to be applied in plants.

The roGFP2-Orp1 H_2_O_2_-sensitive probe is a chimeric construct comprising roGFP2 and Orp1, a yeast oxidant receptor peroxidase which oxidizes the yeast Yap1 transcription factor in the presence of H_2_O_2_. H_2_O_2_ oxidation of the redox active Cys36 in Orp1 forms sulfenic acid,^63^ which then forms an intramolecular disulphide bond with Cys82.^64^ This disulfide bridge is transferred to the reduced roGFP2 cysteines by thiol–disulfide exchange resulting in a conformational remodelling-mediated change in the optical properties of the chromophore. This biosensor has the advantage of being pH-insensitive. roGFP2-Orp1 was first used to monitor H_2_O_2_ dynamics in mammalian cells^64^ but has since been established as a viable H_2_O_2_ sensor in Arabidopsis subcellular compartments capable of revealing spatiotemporal dynamics in H_2_O_2_ signalling.^65,66^

While both the HyPer and roGFP2-Orp1 sensor probes are useful tools to examine H_2_O_2_ signals in plants, they are nevertheless foreign proteins to plant cells. A native probe could be better suited for plant-based ROS-sensing. Native ROS-responsive promoters in plant cells can be coupled to luciferase or fluorescent outputs, such that the signal gives a qualitative readout of cellular ROS. AtHSP70A has been fused with a luciferase (Luc) from *Renilla reniformis* for this purpose,^67^ however, this system requires the supply of exogenous luciferin as a substrate, distribution of which may not be consistent, limiting confidence in ROS estimation. Fusion constructs of native Arabidopsis promoters of RbohD and ZAT12 with GFP have recently been described in Arabidopsis that showed a rapid increase of ROS after exposing plants to different stress conditions.^68^ While the authors reported higher sensitivity and a short response time for the Zat12p-ROS bioreporter, they also saw a rapid decline of the initial high response, even though the half-life of GFP in cells is around 3 h.^69^ This elicits future research to find the exact mechanism behind this rapid decline in GFP to enable improvement of this promising ROS bioreporter.

### 3.3 EPR-based detection of ROS

Electron Paramagnetic Resonance (EPR) spectroscopy can be used to detect unpaired electrons, including ROS (O_2_˙^−^, ˙OH, ^1^O_2_) and NO in biological samples both *in vitro* and *in vivo*. EPR has been applied to date in a range of eukaryotic systems; while mostly used in animal cells, its first application in a biological system was in spinach in 1974, when it was used to quantify chloroplast superoxide production.^70^ Apart from detecting ROS radicals, EPR has also been used to detect stable organic radicals (semiquinone, tyrosyl and carbon-centered radicals) in whole grains, as well as the interaction of paramagnetic transition metal ions Fe(iii) and Mn(ii) with organic and inorganic structures of grains.^71^

#### Spin traps

**Fig. 3:**
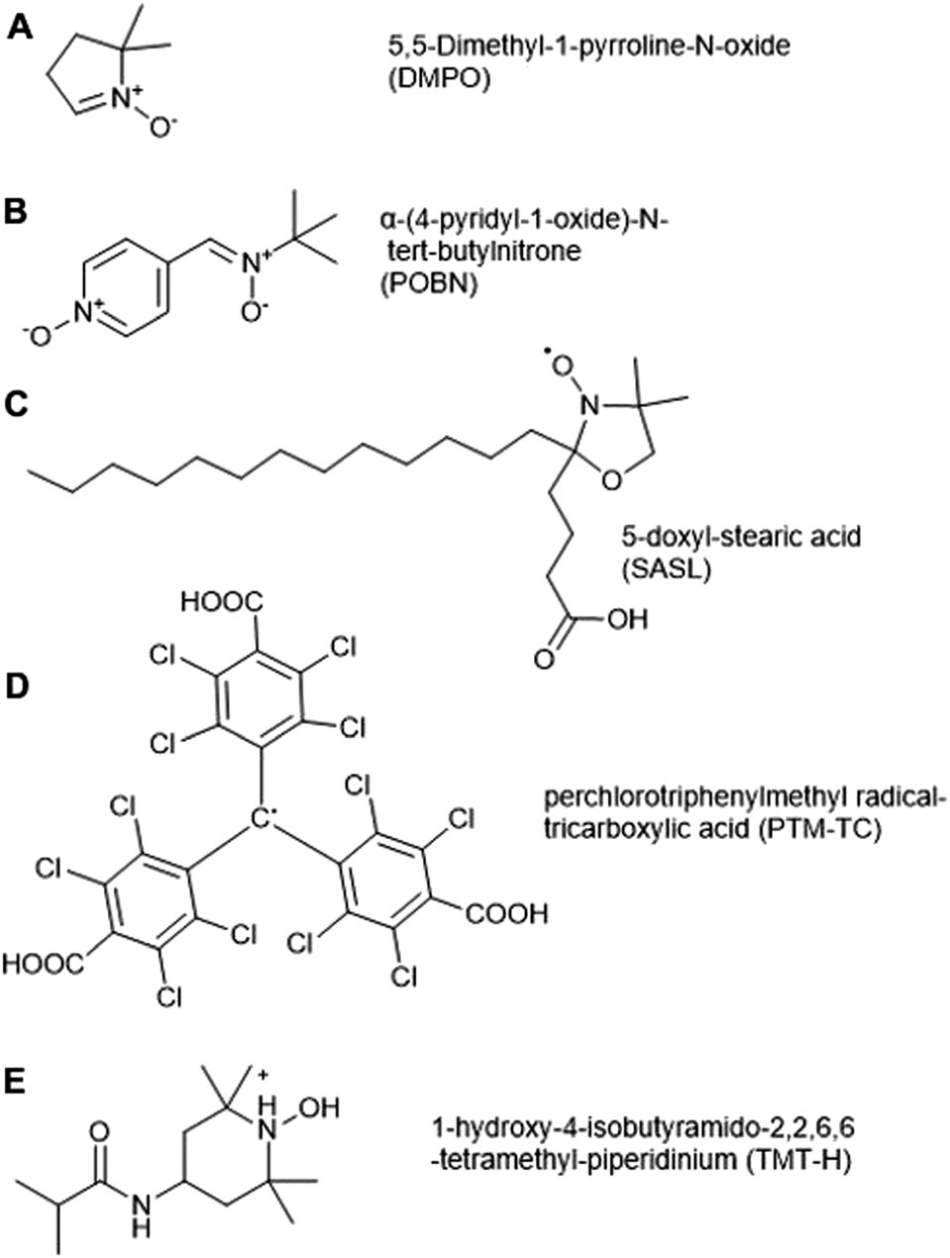
Spin traps and probes used to detect ROS in plant cells. (A) 5,5-Dimethyl-1-pyrroline *N*-oxide (DMPO), (B) α-(4-pyridyl-1-oxide)-*N-tert*-butylnitrone (4-POBN), (C) 5-doxyl-stearic acid (5-SASL), (D) perchlorotriphenylmethyl radical-tricarboxylic acid (PTM-TC), (E) 1-hydroxy-4-isobutyramido-2,2,6,6-tetramethyl-piperidinium (TMT-H).

From the perspective of measuring ROS, the advantage of EPR is that it allows the detection of short-lived highly reactive cellular ROS radicals through the use of spin-traps. A double bond diamagnetic ‘spin trap’ added to a sample can react with free radicals to form comparatively long-lived paramagnetic spin adducts, producing measurable signature EPR signals.^72^ The most commonly used spin traps for ^1^O_2_ are derivatives of the cyclic nitrone 5,5-dimethyl-1-pyrroline *N*-oxide (DMPO, Fig. 3A), which reacts with both ^1^O_2_ and ˙OH to yield distinct spectra. DMPO has been successfully used to detect ^1^O_2_ production from extracted thylakoids^73^ of spinach and derivatives of DMPO have been successfully implemented in plant tissues: DEPMPO, a phosphorylated analogue, forms a stable abduct with ˙OH in the apoplastic fluid of *Zea mays* roots,^74^ while TEMPD (2,2,6,6-tetramethyl-4-piperidone monohydrate) forms an adduct with ^1^O_2_ to form paramagnetic 4-oxo-TEMPO and has been used to follow light induced ^1^O_2_ production in reconstituted membrane of pigment of light harvesting complex II from pea (*Pisum sativum*).^75^

Spin traps Tiron (4,5-dihydroxy-1,3-benzenedisulfonic acid) and 4-POBN (α-(4-pyridyl-1-oxide)-*N-tert*-butylnitrone, Fig. 3B) have been used for O_2_˙^−^ and ˙OH detection respectively, in microsomal and thylakoid membranes, roots, and plant cell suspension culture.^76–79^ There are drawbacks of these two spin traps, for example 4-POBN/OH is converted to a 4-POBN/4-POBN adduct by peroxidase.^80^ Other disadvantages of spin traps are related to their requirement for ethanol as solvent, the slow kinetics of spin trapping and the requirement of high concentrations (10–100 mM) for adequate sensitivity, as well as biodegradation of radical adducts. These factors can limit their application in biological systems.^81^

#### Spin probes

Spin probes, or labels, are an alternative to spin traps for use in plant tissues, and work in the opposite fashion to spin trapping. Intrinsically, spin probes are relatively stable paramagnetic species but are converted to diamagnetic species upon reaction with free radicals, thus resulting in a decrease in their EPR signals. Example of spin probes which have been used in plants are nitroxyl-based probes^82^ such as 5-SASL (5-doxyl-stearic acid, Fig. 3C),^75^ PTM-TC (perchlorotriphenylmethyl radical-tricarboxylic acid, Fig. 3D)^83^ and endogenous cyclic hydroxylamines (CHAs). Usefully, as the SASL probe is lipophilic,^84^ it can easily attach to the lipid phase of membrane vesicles, which is the major site of free radical production in cells. This has been successfully exploited to track total radical production in reconstituted membranes of light harvesting complex II from pea, showing spin decay in the lipid phase following irradiation, but not in the aqueous phase.^75^ In Arabidopsis, the PTM-TC probe was successfully used to quantify and image extracellular O_2_˙^−^ generated in the root because of an apical leaf injury.^85^ This spin probe is specific for O_2_˙^−^, is water soluble, is not modified by other cellular oxidoreductants and requires just a small quantity (10 μM) for a good EPR signal. However, it is cell membrane impermeable meaning its applicability in plants may be limited.^83^ CHA probes are available in two formats; lipophilic TMT-H (1-hydroxy-4-isobutyramido-2,2,6,6-tetramethyl-piperidinium, Fig. 3E) and hydrophilic DCP-H (1-hydroxy-2,2,5,5-tetramethylpyrrolidine-3,4-dicarboxylicacid) are intrinsically EPR silent but become paramagnetic upon reaction with ROS. They react rapidly with O_2_˙^−^ compared to spin traps so can compete with intracellular antioxidants for reaction with ROS. TMT-H has been used in *Oryza sativa* to examine the influence of ethylene on ROS levels in the internode region of the plant.^86^ It has also been used to reveal ROS-mediated signalling networks in post-submergence recovery in Arabidopsis.^87^ However, major disadvantages of the CHA probes are their unspecific interaction with ROS and their autoxidation in the presence of metal ions (Fe^3+^ and Cu^2+^) which requires the use of metal chelators.^88^

Overall, there are limitations of both spin probes and spin traps, but spin probes in particular have generally favourable specificity and temporal properties. EPR spectroscopy is therefore a promising area of development for the analysis of ROS radicals in biological systems, in particular through the use of EPR imaging.^81^ Though its use in plants is limited to date, future research should focus on improving spin probes and traps to increase cell permeability and reduce toxicity. The use of more than one spin trap/probe, if compatible, could also enable dynamic distinction between ROS radicals. Technological innovations in this field, including through reduction in the amount of sample and signal required for detection, mean that adaptive research on plants is highly appealing.

## 4. Measuring the consequences of ROS oxidation: redox status markers

When cellular biomolecules (proteins, lipids, DNA and sugars) are oxidized by ROS/RNS/H_2_S, these modifications reflect the oxidative stress status of the cell^89^ and can be considered as redox markers. In this section, we review the methods that have been developed and adapted in plants to measure these redox markers (see also Table S2, ESI†). Although these are indirect measures of oxidative stress, they nevertheless provide useful information on the general redox status of the cell and have the potential to identify particular ROS-mediated signalling pathways.

### 4.1 Protein oxidation products

Direct modifications of proteins on their amino acid side chains, backbone or conjugated with oxidation products of lipids and carbohydrates are collectively known as oxidative modifications of proteins (Oxi-PTMs). The amino acids that are sensitive targets of ROS/RNS/H_2_S include cysteine, methionine, histidine, tyrosine and tryptophan residues.

#### Cys–Oxi-PTMs

Cysteine (Cys), with its electron-rich sulfur atom, can appear in more than 15 different oxidative modifications.^90^ Among the Cys–Oxi-PTMs, sulfenic acid (RSOH), the first ROS-generated oxidation product of thiols (–SH), can be stabilized through *S*-glutathionylation (–SSG) or disulfide bond formation (S–S). Alternatively, RSOH can be further oxidized to sulfinic (–SO_2_H) acid and to irreversible sulfonic acid (–SO_3_H). Likewise, *S*-nitrosylation (−SNO) and *S*-sulfhydration (−SSH) are generated by RNS and H_2_S, respectively. Besides sulfonic acid, all these Cys–Oxi PTMs can be biologically reduced back to their thiol form with cellular enzymes like glutaredoxin (Grx) or thioredoxin (Trx), as a key mechanism in maintaining redox homeostasis.^91^

**Fig. 4:**
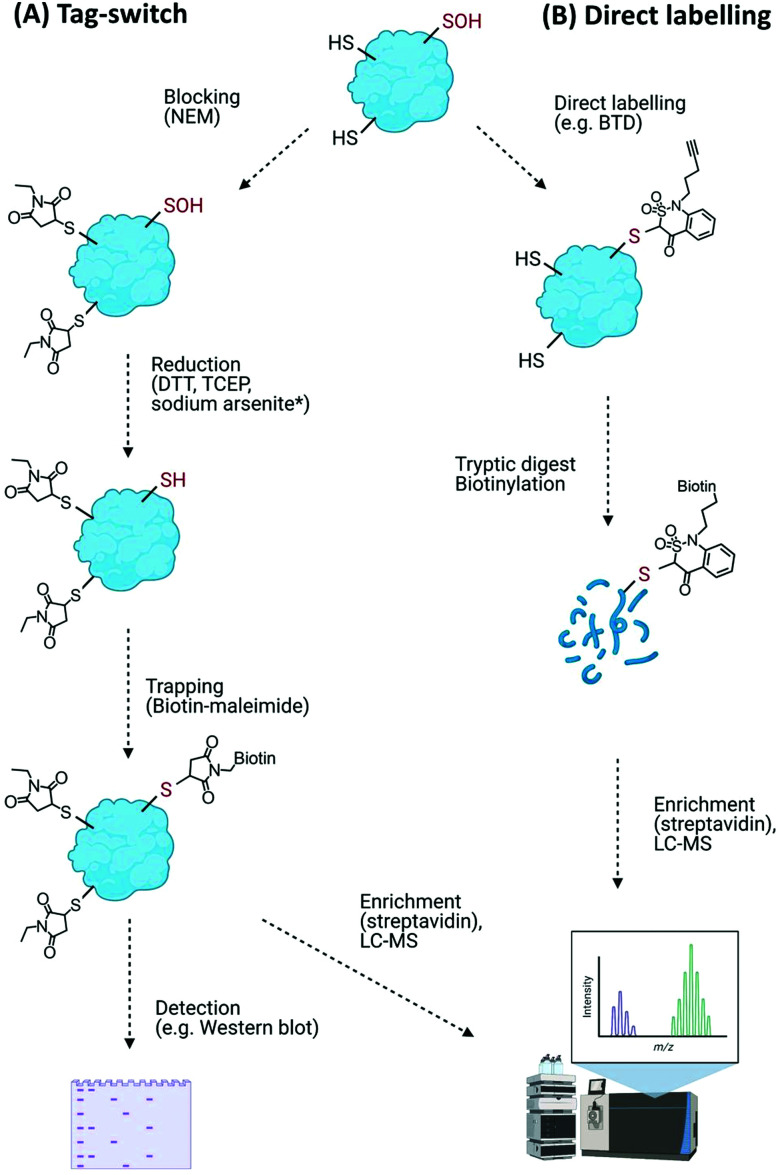
Detection of Cys–Oxi-PTMs using (A) the general tag-switch method whereby thiols are blocked allowing oxidative modifications to be reduced and trapped (* sodium arsenite reduction is specific for Cys–SOH), and (B) direct labelling approach to identify Cys–SOH modifications with specific probes (BTD).

Such transient and reactive modifications make Cys–Oxi-PTMs intrinsically difficult to detect directly, and their identity is often revealed through indirect methods. The most widely used of these methods is differential alkylation, or the tag-switch method (Fig. 4A). This method comprises (i) blocking of free (unmodified) thiols, *e.g.* with *N*-ethylmaleimide (NEM), (ii) reduction of the target modification with general reducing agents (dithiothreitol (DTT), tris(2-carboxyethyl)phosphine (TCEP)) or a selective reducing agent, *e.g.* sodium arsenite to reduce Cys–SOH,^92^ and (iii) trapping of the nascent thiols with detectable tags, *e.g.* biotin-maleimide, as used to detect Arabidopsis chloroplast proteins sensitive to H_2_O_2_-mediated modification.^93^ Similar biotin-switch (BST) methods have been used to identify *S*-nitrosylated proteins involved in a wide range of physiological and stress-related processes in plants.^94^ Modified biotin/tag switch assays have also been developed to identify persulfide modifications arising from *S*-sulfhydration, requiring differentiation between thiol and persulfide groups, *e.g.* by blocking unmodified thiols with the alkylating agents *S*-methyl-methanothiosulfonate (MMTS) or methylsulfonylbenzothiazole (MSBT) before tagging unreacted persulfides with biotin-HPDP.^95,96^

Overall, while broadly useful for monitoring numbers and levels of cysteine oxidation events on proteins, the blocking/reduction/tagging method does not reliably discriminate the nature of the oxidative modification. Moreover, thiol blocking agents can modify off-target basic amino acid residues (*e.g.*, N-termini, lysine and histidine residues) as well as other Cys–Oxi-PTMs (albeit less efficiently). The potential for incomplete blocking, reduction and sample degradation during sample processing are also problematic issues of these types of approach.^91,97^

An improvement on the tag-switch approach for the identification of ROS-mediated sulfenic acid modifications has been achieved through the use of –SOH specific probes for direct trapping or labelling of the modified proteins, affinity enrichment of the targets and identification by mass spectrometry (Fig. 4B). This approach initially used nucleophilic dimedone for –SOH labelling,^98^ however, dimedone lacks a functional group which limited its application for subsequent proteomic study. Dimedone-functionalized probes were therefore developed for sulfenylated protein identification, including addition of biotin, azide and alkyne groups.^99,100^ Using such dimedone probes together with mass spectrometry has enabled identification of 226 sulfenylated proteins in H_2_O_2_-treated Arabidopsis cells^100^ and 11 sulfenylated proteins in H_2_O_2_-stressed Arabidopsis seedlings.^101^

While these probes could detect overall levels of SOH in plant cells, the identification of the site of modified cysteines became possible with the use of a more reactive SOH reactive probe, BTD (1-(pent-4-yn-1-yl)-1*H*-benzo[*c*][1,2]thiazin-4(3*H*)-one 2,2-dioxide) with a ‘clickable’ handle for biotinylation of labelled fragments (Fig. 4B). Combined with subsequent enrichment and MS analysis, BTD has enabled both identification and quantification of redox-sensitive sites in Arabidopsis proteins.^102^ As well as this chemical probe, a non-invasive SOH-trapping biosensor, the plant-optimized and affinity-tagged yeast AP-1 (YAP1C), has been exploited to capture and identify sulfenylated proteins in Arabidopsis, including characterisation of the sulfenome at a subcellular level.^103,104^ These probes are currently revealing the extent to which overall cysteine oxidation is impacted by ROS stress; it will be interesting to determine whether these modifications initiate specific signalling events, *e.g.* by modulating the stability of proteins with N-terminal cysteine residues *via* the Cys/Arg branch of the N-degron pathway.^105^

A range of techniques has been used to detect other Cys modifications in plant and other systems. Proteomic approaches dedicated to detection of disulfide bonds in plants are based on thioredoxin (Trx)-affinity chromatography, using immobilized monocysteinic Trx as a ligand.^106,107^*S*-Glutathionylated proteomic analyses in plants use 35S-radiolabelled Cys, biotinylated glutathione (GSH-biotin/GSSG-biotin), or biotinylated reduced glutathione ethyl ester (GEE-biotin).^108–110^

Sulfinylated proteins (SO_2_H) can be detected by mass spectrometry,^111^ antibody-based approaches^112^ and chemical probes including aryl-nitroso compounds,^113^ maleimide compounds,^114^ and electrophilic nitrogen species-based –SO_2_H probes including NO-Bio^113,115^ and DiaAlk.^116^ These have been applied to sulfinylation proteomics, but none of these methods has yet been reported *in planta*. Similarly, the irreversible sulfonic acid (SO_3_H) modification has not been detected in plants. Poly-arginine (PA)-coated nano-diamond high-affinity probes have been used to detect SO_3_H modifications in mixtures of proteins, but due to competition with phosphorylated peptides the number of detected –SO_3_H-containing proteins is limited.^117^

#### Other Oxi-PTMs in proteins

Methionine sulfoxide (MetSO) is formed through the reaction of ROS with the thioether group (R–S–R′) of methionine and is susceptible to further oxidation to methionine sulfone.^118^ This modification has been identified in Arabidopsis through the use of immobilized AtMSRB1, a methionine sulfoxide reductase,^119^ and site specific quantification of methionine oxidation has also been achieved in Arabidopsis using a combined fractional diagonal chromatography (COFRADIC) approach.^120^ Recently, an *in vivo* site centric quantification of methionine oxidation was reported in human cells using the isotopic labeling (H_2_^18^O_2_) of unoxidized methionine residues,^121^ however, this approach has not yet been reported in plants.

The tryptophan radical can react with ROS to form tryptophan hydroperoxide, which then rearranges to *N*-formylkynurenine (NFK) and kynurenine.^122^ Detection measures used in plants are based on mass spectrometry: oxidation-sensitive NFK modified proteins in potato tubers and rice leaves have been identified using Blue Native PAGE or 2D electrophoresis coupled with LC-MS/MS,^123^ and tandem mass spectrometry has identified NFK modification of specific tryptophan residues in proteins of the Photosystem II complex.^124^

Protein carbonylation is the irreversible oxidative modification of side chains of proline, histidine, arginine, lysine and threonine. The result is the formation of reactive carbonyls and usually leads to the inactivation of protein function through unfolding and an increased susceptibility to degradation;^125^ global levels of carbonylation can be detected using a number of assays. Derivatisation of carbonyl groups with 2,4-dinitrophenylhydrazine (DNPH) allows spectroscopic detection, or immunodetection using DNPH-specific antibodies and subsequent tandem MS can identify specifically oxidized proteins, *e.g.* in rice leaf mitochondria exposed to H_2_O_2_.^126^ A fluorescence-based method using fluorescein-5-thiosemicarbazide (FTC) has also been used to detect an increase in carbonylated protein levels during natural senescence of the wheat fag leaf.^127^

### 4.2 Lipid oxidation

Oxidative damage of the fatty acids of cell membranes, lipoproteins, and other lipid-containing structures is collectively termed as lipid peroxidation and is another marker of altered redox status in plants.^128^ The reactive carbonyl species resulting from lipid oxidation comprise malondialdehyde (MDA), 4-hydroxy-(*E*)-2-nonenal (HNE) and acrolein; these originate from enzymatic and non-enzymatic degradation of lipid hydroperoxides, the primary oxidized products of polyunsaturated fatty acids.^129^ Techniques which have been used to detect these modifications include the FOX2 (ferrous oxidation in xylenol orange) assay,^130^ chemiluminescence based on luminol oxidation by hydroperoxide^131^ and a thiobarbituric acid-reactive substances (TBARS) assay, which is an index of general lipid peroxidation^132^ and has been used to localize MDA pools throughout the body of Arabidopsis.^133^ More specific methods for detection of lipid oxidation products in plants have also been emerging ranging from antibody based detection,^134^ HPLC followed by MS/MS,^135^ derivatization, chromatographic separation and MS^136^ and *in vivo* detection using fluorescence microscopy.^133,137^

### 4.3 Sugar oxidation

Water-soluble carbohydrates comprising disaccharides (sucrose, trehalose), raffinose family oligosaccharides (RFOs) and fructans occupy a central position in cellular redox balance and are involved in plant signalling networks regulating stress and defence responses; their accumulation in transgenic plants suggests they play an antioxidant role in oxidative stress.^138^ Plant-derived sugars can also, at least *in vitro*, scavenge hydroxyl radicals during Fenton reactions with Fe^2+^ and hydrogen peroxide, leading to formation of less toxic sugar radicals,^139^ suggesting an additional antioxidant role. Measuring soluble carbohydrate levels may therefore give an indication of oxidative stress and can be simply, though crudely achieved using a phenol–sulfuric acid colorimetric method.^140^ More accurate determination of oxidised sugar levels in Arabidopsis and barley has also been achieved using mass spectrometric proteomic (MS and MS/MS) techniques.^141^

### 4.4 Nucleic acid oxidation

ROS/RNS can generate a large number of oxidative modifications of nucleic acids, including 8-hydroxydeoxyguanosine (8-OHdG) in DNA, 8-hydroxyguanosine (8-OHG) in RNA and 8-nitroguanine (8-NO_2_-G). Nucleic acid oxidation by ROS plays important biological roles, *e.g.* in release from dormancy in seeds,^142^ but is also used as a marker of oxidative stress and 8-OHdG can be detected using antibody-based immunoassays, which have been exploited to observe increased DNA damage in Arabidopsis seeds lacking the base excision-repair enzyme AtOGG1.^143^ Chromatographic (HPLC) methods have also be used to measure 8-OHdG in cryopreserved currant species exposed to temperatures of −20 and −196 °C.^144^ More refined chromatography/mass spectrometry methods coupled with immunoaffinity purification or derivatisation have been used to detect oxidized bases in animal samples, but have not yet been applied in plants.

## 5. NADPH and NADH in the redox landscape

The pyridine nucleotides NADH and NADPH are tightly associated with redox metabolism. NADPH is the ultimate donor of reducing power for the majority of ROS-detoxifying enzymes^145^ as well as providing the electrons for generation of O_2_˙^−^ radicals by RBOH enzymes.^146^ The function of NAD^+^ is to act as an electron acceptor during the oxidation of substrates; the NADH generated is then used to make ATP in the mitochondrial electron transport chain. Dysfunction in the balance of redox metabolism can cause changes in concentrations of NAD(H). For example, hypoxia causes accumulation of NADH due to a lack of O_2_ as a terminal electron acceptor.^147^ Therefore, NAD(P)(H) are critical markers of redox metabolism providing information that can explain both the production and consumption of ROS. Although NAD(P)(H) can be extracted from cells and analysed by enzymatic or HPLC methods, more accurate and relevant information can be gained from *in vivo* approaches, described here and in Table S3 (ESI†).

### 5.1 Autofluorescence

NAD(P)H is intrinsically fluorescent (excitation 340 nm, emission 445 nm) and this can be exploited to measure total NAD(P)H concentration *in vivo*.^148^ However, weak signals and lack of specificity between NADPH and NADH limit the usefulness of this method. More sophisticated measurement exploits the difference in fluorescence lifetime of free and protein-bound NAD(P)H to gain more information.^149^ Fluorescence lifetime imaging (FLIM) can even allow estimation of absolute NAD(P)H concentration in live cells,^150^ although this is yet to be applied to plants.

### 5.2 Chemical probes

Chemical probes can be used to improve the limit of detection for NAD(P)H beyond that achieved using autofluorescence. Probes are typically based on a recognition unit that takes advantage of chemical reduction by NAD(P)H by linking compounds such as quinone, resazurin quinolinium and pyridine to fluorophores such as fluorescein, rhodamine and rhodol (see Sun *et al.*^151^ for a recent review). Whilst many of these probes have been established and tested in mammalian systems, often studying cancer,^152^ there has been very little application to plants, potentially due to issues with cell wall and tissue permeability. A major limitation to current probes is their reliance on chemical reduction by NAD(P)H, leading to potential toxicity as cellular NAD(P)H is consumed. This mechanism also rules out detection of the oxidised forms, NAD^+^ and NADP^+^, which can be critical for calculating ratios and redox potentials, or investigating the important signalling functions of pyridine nucleotides.^153^ Furthermore, whilst some probes may show slight preference for NADPH or NADH, in general they are non-specific, and report only on total NAD(P)H levels. The major advantage of chemical probes, however, is the avoidance of needing to genetically modify plants to use them, but when specific and dynamic measurement is required, genetically encoded protein-based sensors are a more useful tool.

### 5.3 Fluorescent protein sensors

Fluorescent reporter proteins can be specific to the oxidised and reduced forms of NAD(P)H and can provide additional subcellular specificity through targeting strategies. Many different NAD(P)(H) sensors exist (reviewed by Kyere-Yeboah *et al.*^154^) but only SoNar,^155^ iNap^156^ and Peredox^157^ have been expressed to date in plants.^158,159,203^ All three sensor proteins are based on the bacterial Rex NADH binding protein from *Thermus aquaticus* fused to either cpYFP or cpT-Sapphire, with SoNar and Peredox sensitive to the NADH : NAD^+^ ratio^155,157^ whereas iNap detects NADPH alone.^156^ The fluorescent sensors have different binding affinities for their substrates and careful selection of sensor variants is required to ensure physiological changes can be detected. Four different versions of iNap exist, with a range of NADPH binding affinities from 0.3–120 μM;^156,158^ two versions of Peredox have been developed with an NADH binding affinity of 1.2 μM and 31.4 μM respectively (in the presence of 500 μM NAD^+^).^159^

Peredox has already been applied to explore the role of the nucleotide transporter NDT in stomatal development,^160^ the importance of malate dehydrogenase for redox shuttling during photosynthesis^161^ and a possible link between pathogen elicitation and the cytosolic NADH : NAD^+^ ratio.^159^ SoNar and iNap have been used together with subcellular pH and ATP sensors to investigate photorespiration, supporting the hypothesis that reductant in excess of the capacity of the mitochondrial electron transport chain is produced during photosynthesis.^158^ Although SoNar and iNap have successfully been expressed in the cytosol, chloroplasts and peroxisomes, no sensor for NAD(P)(H) has yet been localised to plant mitochondria. In contrast, chemical probes for NAD(P)H are able to penetrate the mitochondria in human cells^162^ and the NADPH biosensor iNap has been successfully expressed in the mitochondria of human cells.^163^

Improvements could be made with greater diversity of cellular targeting of chemical probes and biosensors, *e.g.* Apollo-NADP^+^ for NADP^+^ or LigA-cpVenus for NAD^+^ detection.^164^ Calibration of sensors can also be challenging, but progress in this area could help improve the quantitative nature of measurements so they can be integrated into kinetic or thermodynamic models of plant metabolism.^203^ Application of advanced FLIM methodologies to plants or new chemical probes specific to NADPH or NADH might also help provide this information in future.

## 6. Measurement of ROS scavengers: ascorbate and glutathione

Ascorbate and glutathione are prevalent small molecule antioxidants that play a role in redox regulation through ROS ‘scavenging’, *i.e.* ready reaction with H_2_O_2_ and O_2_˙^−^. These oxidation events are tightly coupled to each other and to NADP^+^ oxidation *via* the enzymatically-controlled ascorbate–glutathione cycle. Both ascorbate and glutathione levels can therefore provide an insight into the redox status of the cell and may also act as a signalling ‘hub’ for downstream effects.^165^ Robust methods to measure these anti-oxidants, including discrimination from their oxidised forms, is therefore necessary; current protocols are summarised here and in Table S4 (ESI†).

### 6.1 Ascorbate

Ascorbate is distributed in different subcellular compartments, performing diverse functions including cellular redox defence and signalling, growth and development.^166^ Although ascorbate is ubiquitously found across subcellular locations, determining the exact concentration in different compartments is still technologically challenging. A number of methods have been used in plant tissue or organ-based extracts, including titration with oxidants to form coloured salts (dichlorophenol indophenol and potassium iodate or borate), spectrophotometric measurement of ascorbate depletion on addition of ascorbate oxidase and voltammetric titration.^167^ Improved sensitivity is achieved by using HPLC, which has been used to determine total ascorbate in horticultural products^168^ as well as to demonstrate fluctuations of ascorbate levels in different organs and growth phases of Arabidopsis.^169^ Apart from the voltammetric approach, these methods require sample preparation procedures that increase the risk of ascorbate oxidation. Moreover, as all these estimations are from collections of cells, the interpretation of ascorbate level estimates at the individual cellular level is missed. Nevertheless, these accessible approaches have been widely used.

Subcellular estimation of ascorbate levels uses different approaches. Histochemical labelling with silver nitrate enables detection by microscopy, with a degree of specificity if conducted under cold and acidic conditions.^170^ Anti-ascorbate antibody labelling coupled with gold nanoparticle labelled secondary antibodies has quantified ascorbate levels in different cellular sub-compartments of Arabidopsis and tobacco plant leaves^171^ when coupled with high-resolution transmission electron microscopy (TEM); whilst a promising method for visualising ascorbate, the primary antibody cannot distinguish among ascorbate, dehydroascorbate (DHA) and monodehydroascorbate. Finally, *in vivo* real time imaging of ascorbate has recently been achieved using a selective fluorescent probe, made of silicon phthalocyanine (SiPc) and two 2,2,6,6-tetramethyl-1-piperidinyloxy (TEMPO) radicals, albeit in an animal model^172^ and yet to be tested in plants.

### 6.2 Glutathione

The tripeptide glutathione is a major non-enzymatic component of the cellular redox repertoire, and significantly contributes to the maintenance of cellular redox homeostasis. Glutathione can be reduced (GSH), oxidized (GSSG), or conjugated to glutathionylated proteins. *In vitro* and *in vivo* estimation of glutathione can be performed using Ellman's reagent, 5,5′-dithio-bis(2-nitrobenzoic acid) (DTNB), which oxidises GSH to form the yellow derivative, 5′-thio-2-nitrobenzoic acid, measurable by absorbance at 412 nm. GSSG is estimated *via* coupling an enzymatic assay consisting of glutathione reductase (GR) and NADPH to reduce GSSG to GSH prior to reacting with DTNB. This method is not specific to measurement of glutathione, as DTNB also reacts with other protein and non-protein cellular thiols.^173^ Nevertheless, as GSH is present at such high concentrations compared to other thiols, it provides an approximate estimate of cellular glutathione and has been used in a number of significant studies to reveal glutathione cellular function, *e.g.* nuclear recruitment of glutathione during the cell growth cycle.^174^

Chromatographic estimation of glutathione can be performed using HPLC combined with derivitisation of GSH with monobromobimane (MBB)^175^ or *O*-phthaldialdehyde (OPA).^176^ Capillary electrophoresis^177^ and LC-MS^178^ have also been used for glutathione estimation and identification of glutathione-conjugates in plant extracts and fractionated organelles. However, all *in vitro* methods have their own drawbacks and limitations, notably their limited specificity and alteration of the glutathione pool during extract preparation and mixing from subcellular compartments.

*In situ* detection and estimation of glutathione, particularly at the subcellular level, provides new insights into its role in responses to cell stress. Monochlorobimane and monobromobimane fluorescent dyes have been used to report glutathione in trichoblast (root hair cells), atrichoblasts, and nuclei and cytosol of different plant cells.^179–181^ However, these dyes are problematic due to their cellular toxicity, nonselective thiol labelling, and inability to infiltrate chloroplasts.^182^ Similar to ascorbate, immunogold labelled glutathione can be visualised with transmission electron microscopy, however, GSH and GSSG cannot be distinguished, and cells must necessarily be fixed for visualisation. The fluorescent Grx1-roGFP2 biosensor (Section 3.2), fused to human glutaredoxin, can measure the redox potential of the glutathione pool without many of the problems associated with dyes, and can be quantitative if appropriately calibrated.^42^ FLIP-G (fluorescence indicator protein for glutathione), a recently developed genetically encoded FRET based sensor, was successfully used in bacterial and yeast systems for real-time monitoring of glutathione;^183^ it remains to be tested in plants but could lead to new insights in glutathione research including with potential for sub-cellular targeting.

## 7. Measuring oxygen/hypoxia

A strong indicator of the propensity for redox stress in a plant cell or tissue is the amount of oxygen available. While oxygen is not a ROS, hypoxia (reduction in oxygen availability) can result in elevated ROS: oxygen is required as the terminal electron acceptor in oxidative phosphorylation so hypoxia or anoxia can lead to a greater likelihood of electron ‘leakage’ from the mitochondrial membrane resulting in ROS formation. Furthermore, O_2_ fluctuations (*e.g.* recovery after submergence) may result in damaging ROS bursts.^184^ Measuring O_2_ levels can therefore be complementary to studies of ROS and redox markers and we discuss here current methods employed to achieve this in plants (see also Table S5, ESI†).

### 7.1 Physical probes

Until now, the method used most widely in the plant field for measuring O_2_ concentrations in tissues is the Clark-type electrode. These were developed over half a century ago to measure O_2_ in solution^185^ and comprise an O_2_-permeable membrane, *e.g.* Teflon, covering a platinum cathode. O_2_ in the test solution diffuses to the electrode where it reacts with electrons to form water. A change in current measures the amount of O_2_ diffusing to the cathode. These electrodes have been used to measure O_2_ concentrations in a variety of plant cell and tissue suspensions, and in recent decades, micro-electrodes have been used to measure O_2_ concentrations in plant tissues including chickpea roots, growing potato tubers, and castor plant stems.^186–188^ The best spatially resolved signal to date has been achieved with a miniaturised electrode whose 3 μm tip has been used to define O_2_ concentrations to 10 μm resolution. This enabled definition of a discrete hypoxic zone in Arabidopsis shoot apical meristems and the identification of a new mode of hypoxia-triggered developmental signalling.^189^ Clark electrodes have therefore contributed a great deal to understanding O_2_ distribution in plants and are relatively easy to manipulate in tissue. Their disadvantage is that they are necessarily invasive, they consume O_2_ (so can detrimentally deplete O_2_ supply) and they can only measure O_2_ concentration in one physical location at a time meaning it is not easily possible to visualise O_2_ distribution, although careful manipulation (even with the miniaturised electrode) can reveal cross-sections of O_2_ gradients across tissues.

Another physical method of measuring O_2_ concentration is through the use of probes that exploit O_2_-quenching of molecular luminescence. These constitute optic fibres coated in an indicator molecule, commonly phosphorescent Pt(ii)– and Pd(ii)–porphyrins. Like the Clarke electrodes, these probes have also successfully measured gaseous and dissolved O_2_ concentrations in a range of plant tissues including generation of O_2_ maps in seeds^190^ and measurement of O_2_ dynamics in deepwater rice and seagrass.^191,192^ However optic fibres, while they don’t consume O_2_, encounter similar challenges as Clarke-type electrodes, in that they can be invasive and physically damaging. The signal can also be impacted by chlorophyll fluorescence and incident light. Soluble luminescent indicators could be very useful, particularly if they could enter cells to measure intracellular O_2_ concentrations. Towards this aim, Pt(ii)–tetra-pentafluorophenyl-porphyrin has been encapsulated in polystyrene microbeads and injected into plant cells where, despite an overlap in fluorescent spectra from the probe and chlorophyll, multi-frequency phase modulation allowed determination of O_2_ concentration, albeit without subcellular resolution.^193,194^ To avoid the requirement for micro-injection, nanoparticles could be a potential route for future delivery of luminescent O_2_ probes. Gold nanoparticles have indeed been shown able to enter plant tissue *via* roots and translocate to stems and leaves, entering cells by active transport mechanisms.^195^ Overall, however, this method of O_2_ measurement remains to be advanced in the plant field.

### 7.2 Chemical probes

Fluorescent and luminescent chemical probes and dyes have been extensively investigated for use in measuring tissue and tumour hypoxia in humans (reviewed in ref. 196). None of these has yet been successfully applied in plant cells, likely due to issues including cell entry, toxicity and interference with intrinsic cellular fluorescence. However, continued development of O_2_-probes in the mammalian tumour biology field may yield success in plant cells. One interesting area of development is the use of hypoxia-triggered fluorescent probes. For example, an azide-conjugated fluorophore is activated when the azide group is bioreduced in hypoxic conditions, enabling detection of hypoxia in hepatic and colorectal cancer cells and spheroids.^197^ A similar probe, based on bioreduction of 4-nitrobenzene-conjugated resorufin, has been used in bacterial cells to detect hypoxia,^198^ demonstrating its ability to enter ‘walled’ cells. Application of such probes could be successful in plant cells and tissues, enabling O_2_ detection without the need for physical probe insertion or genetic manipulation. However, their ability to detect specific O_2_ concentrations or O_2_ fluctuations is not clear.

### 7.3 Genetically-encoded O_2_-measurement strategies

**Fig. 5:**
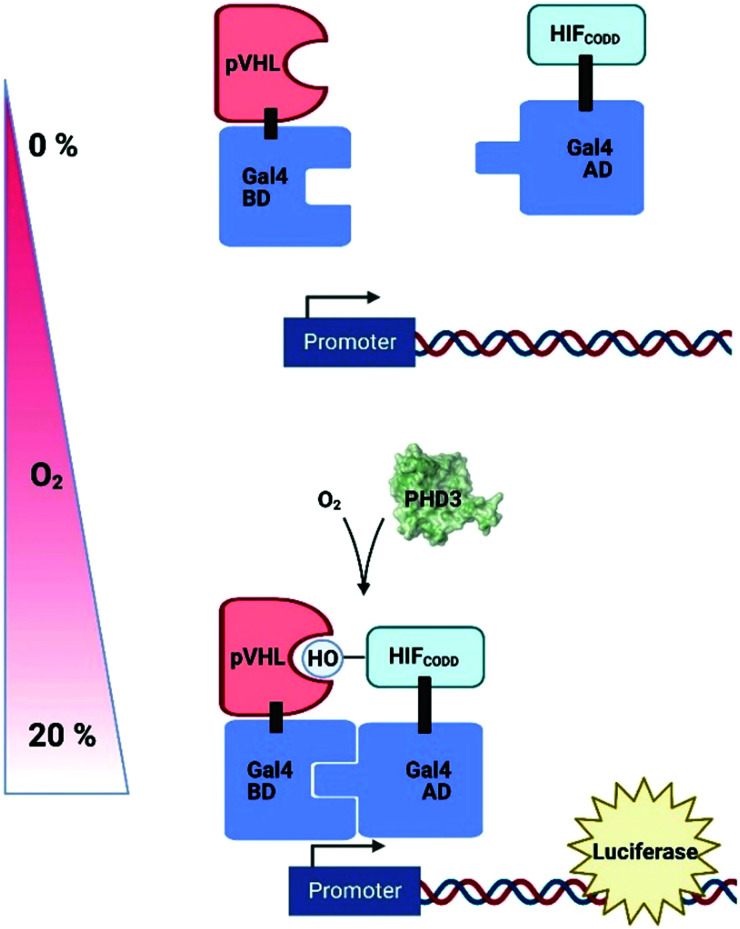
A synthetic O_2_-sensing cassette in plants exploiting human prolyl hydroxylase 3 (PHD3) O_2_-dependent hydroxylation of HIF (Hypoxia Inducible Factor); pVHL binding of hydroxylation site brings together Gal4 binding domain (BD) and activating domain (AD) to promote expression of luciferase. pVHL, Von Hippel Lindau protein; CODD, C-terminal oxygen dependent degradation domain.

Genetically-encoded strategies have been developed in plant cell systems in an attempt to measure intracellular O_2_ levels in a non-invasive fashion; these have exploited existing systems of hypoxia-dependent transcription to produce reporter molecules. A synthetic O_2_-sensing cassette has been engineered into both Arabidopsis protoplasts and plants which uses the human oxygen-sensing enzyme Prolyl Hydroxylase 3 (PHD3) along with other components of the human hypoxic response system to regulate levels of luciferase (Fig. 5).^199^ In normoxic conditions, PHD3 hydroxylase activity results in binding of a hydroxylated HIF (hypoxia-inducible factor) protein and pVHL, bringing together Gal4 activation and binding domains to promote luciferase expression. In hypoxia, however, HIF is not hydroxylated and no reporter molecule is produced. While this system was complex to prepare and slow to respond to changes in O_2_ concentration, the authors were nevertheless able to demonstrate dynamic sensitivity to different O_2_ concentrations and, importantly, the signal was reversible.

The use of genetically-engineered fluorescent proteins for O_2_ measurement is challenging, particularly in plant cells,^200^ because of the confounding challenges of chlorophyll fluorescence and the fact that commonly used GFP-derivatives require O_2_ to form a chromophore. Recently, however, an O_2_-independent green fluorophore (UnaG, previously used in tumour cells^201^) was shown to fluoresce in anoxic Arabidopsis protoplasts. When the expression of both UnaG and the O_2_-dependent fluorophore, mCherry, were coupled to a hypoxia-responsive promoter element, the ratio of emission from the two fluorophores could be correlated to O_2_ concentration in hypoxic *Nicotiana benthamiana* tissues between 0–5% O_2_.^202^ This and the PHD3-based biosensor both rely on translation of a reporter molecule in response to O_2_ availability, so neither will show rapid dynamics, but they have potential for subcellular targeting and could therefore provide important information on cross-cellular O_2_ gradients, something that is lacking with current technologies.

## 8. Conclusions

The extensive literature we have cited in this review, as well as significantly more which we have been unable to cite for space reasons, demonstrates that a wide range of techniques is currently in use to measure ROS and redox markers in plant cells and tissues. ‘Classic’ biochemical techniques, such as those used to measure glutathione and ascorbate, tend to rely on cell lysis and staining or detection in an *ex vivo* environment and are prone to errors due to stress inherent in the methodology. Other methods broadly fall into the categories of biosensors (detected by luminescence or fluorescence) and chemical probes (detected by similar outputs as well as mass spectrometry when used with ‘handles’ such as biotin). Both types of method have advantages and disadvantages: Biosensors are dynamic and often specific, but their signals can be affected by cellular conditions such as pH and suitable controls are needed to account for expression levels. Biosensors must also be introduced *via* genetic engineering, so are only applicable in transformable species. On the other hand, chemical probes are, in principle, quickly applicable to any plant. However, they must be able to enter the cell or tissue and access the locations of interest; permeability, transport and stability issues are therefore important. Chemical probes are often less specific than biosensors, prone to react with multiple oxidising species. Arguably, probes that are designed to identify oxidised proteins, *e.g.* Cys–Oxi-PTMs, are more specific for their target modifications; however, experimental design needs careful consideration to maintain the integrity of each modification through the process. Overall, current tools and techniques for ROS and redox marker measurement require careful controlled use and shouldn’t be over-interpreted.

There is significant scope for an improved and expanded toolkit; while it is not yet possible to discriminate different ROS signals with the temporal and spatial resolution required to really understand their specific cause and effects, recent developments show continuing promise. Intracellular fluorescent biosensors, despite their limitations, are favourable with respect to specificity and subcellular targeting. Meanwhile nanoprobes have excellent potential when it comes to combining these properties with versatility of target plant/tissue and ease of application. There is also considerable scope for ROS-activated chemical probes to be developed with improved characteristics. Overall, continued investment is required to generate advanced chemical and biological probes for ROS and redox measurement in plants; particularly desirable are direct ROS-sensing probes that can rapidly elicit a response in order to understand cellular ROS dynamics. We encourage the chemical biology community to take on this challenge!

## Author contributions

All authors contributed to writing and editing the manuscript.

## Conflicts of interest

There are no conflicts to declare.

## Supplementary Material

CB-002-D1CB00071C-s001
