# Supplementary material for: Measuring ROS and redox markers in plant cells
Source: RSC Chem Biol. 2021 Jun 29;2(5):1384–401. doi: 10.1039/d1cb00071c (PMC8495998; doi:10.1039/d1cb00071c)
Supplement: CB-002-D1CB00071C-s001 [file CB-002-D1CB00071C-s001.pdf]

Electronic Supplementary Material (ESI) for RSC Chemical Biology.
This journal is © The Royal Society of Chemistry 2021

Measuring Redox Markers in Plant Cells

S Akter,[a,b] MS Khan,[a,b] E Smith[c] and E Flashman [a]

Supplementary Information

Contents **Page**

Supplementary Table S1: Summary of methods used to directly detect and quantify ROS. 2

Supplementary Table S2: Summary of methods used to detect and quantify redox markers. 5

Supplementary Table S3: Summary of methods used to detect and quantify NAD(P)/H 7

Supplementary Table S4: Summary of methods used to detect and quantify ROS scavengers 8

Supplementary Table S5: Summary of methods used to detect and quantify O_2 9

References 10

Supplementary Table S1. Summary of methods used to directly detect and quantify ROS.

Target	Method/Probe	Quantitative/ qualitative	Ratiometric?	Examples of plant species	Spatial and/or temporal resolution	Applications	Limitations
**Chemical Probes**							
**$O_2^{\bullet-}$**	Tetrazolium dyes	Semi-quantitative (requires extraction and chromatographic measurement)	No	Tobacco suspension cells, strawberry plant leaves	Tissue-level resolution	Measuring hypersensitive response of tobacco to parasite infection [1], quantification of ROS in strawberry plants exposed to different stress conditions [2].	Non-specific to $O_2^{\bullet-}$, affected by O_2 availability and can generate $O_2^{\bullet-}$
	Dihydroethidium	Semi-quantitative (requires extraction and chromatographic measurement)	No	Pea and rice seedlings	Cytosolic	Investigating silver nanoparticle toxicity in rice [3] and Cd toxicity responses in pea [4]	Specificity requires chromatographic verification of oxidation product. Can react with other cellular components
	MitoSOX	Quantitative	No	Arabidopsis	Mitochondrially-targeted but extraction may be required	Salicylic acid induced ROS generation and signaling pathway [5]	Specificity requires chromatographic verification of oxidation product
**H_2O_2**	Cerium chloride	Quantitative with transmission electron microscopy	No	Lettuce leaves	5h-8h incubation required; targets cell wall	Hypersensitivity in response to bacterial infection [6]	Requires long incubation times and specialized equipment
	3,3'-Diaminobenzidine (DAB)	Qualitative	No	Arabidopsis leaves, tomato fruit, roots and stems	4h-5h incubation required; shorter (15s) with tissue printing[7]	Peroxidase-dependent oxidative burst in Arabidopsis immunity [8]; H_2O_2 localization in large and thick plant organs; stem, roots and fruits [7].	Requires long incubation time and peroxidase activity
	Luminol (Chemi-luminescence)	Quantitative for relative changes	No	Garden cress, rosehip, coltsfoot and English plantain.	Fast detection (seconds)	Abiotic stress (salt, drought, cold, and heat) response of the antioxidative system [9]; Antioxidants in herbal extracts [10]	Signal quenching by other cellular components
	Amplex Red	Quantitative for relative changes	No	Arabidopsis and tobacco leaves	Extracellular	Detection of extracellular H_2O_2 in Arabidopsis [11] and tobacco leaves [12]; H_2O_2	Cell-impermeable, photochemical oxidation is

						production in response to heavy metals[13]	possible, may cause cellular stress
	Dihydrofluorescein and related molecules	Qualitative and quantitative	No	Tobacco, poppy plants and Arabidopsis	Intracellular; fluorescence develops within minutes of H_2O_2 exposure.	ROS generation during pollen tube development [14]; Self-incompatibility response of poppy [15]; plant pathogen interaction[16]; role of H_2O_2 in ethylene-induced stomatal closure.[17]	Can react with other ROS, susceptible to photooxidation and photobleaching
	Boronate probes	Qualitative	No	Arabidopsis	Intracellular using confocal microscopy	Response to bacterial infection [18]	Can also be oxidized by HOCl and $ONOO^-$
	Single-walled carbon nanotube	Quantitative (ratiometric)	Yes	Arabidopsis, lettuce, arugula, spinach, strawberry, sorrel.	Spatial resolution at tissue level (leaf regions). Temporal resolution limited by camera frame rate of 2 frames per second; quenching 1-2 mins	H_2O_2 fluctuations post wounding and light stress [19]	Not (yet) commercially available
	Hybrid of silicon oxide quantum dots and silver nanoclusters	Quantitative with optimisation	Yes	Lettuce	Spatial resolution at tissue level (leaf regions).	Wound-induced H_2O_2 formation in lettuce [20]	Fluorescence signal overlaps with chlorophyll fluorescence
**$^1O_2$**	DanePy	Quantitative for relative changes	No	Broad bean leaves, Arabidopsis	Thylakoid membranes, Arabidopsis chloroplast	Photoinhibition of broad bean leaves [21]; Excess photosynthetically active radiation in Arabidopsis [22]	ROS mediated quenching of fluorescence
	Singlet Oxygen Sensor Green (SOSG)	Quantitative for relative changes	No	Arabidopsis	Spatial resolution at tissue level; ~20 mins required for signal detection	High light-dark treatment, wounding and herbicide (DCMU) treatment [23].	UV photobleaching and high photosensitivity. Cell impermeable.
**Fluorescent Biosensors**							
**Redox status**	roGFP1 and roGFP2	Quantitative and qualitative	Yes	Arabidopsis	Cytosol, mitochondria, ER, peroxisomes	Measurement of glutathione redox potential in the cytosol [24]; drought [25] and salinity stress[26]	Global cellular redox status only, lack specificity, requires transformation
**H_2O_2**	HyPer and roGFP2-Orp1	Qualitative and quantitative	Yes	Tobacco, Arabidopsis, legume-rhizobium symbioses	Chloroplast stroma, cytosol, nuclei and mitochondria; rhizobium nodules	Photosynthesis-dependent H_2O_2 transfer in *Nicotiana* [27]; monitoring *in vivo* H_2O_2 dynamics during elicitor-	pH-sensitivity needs to be carefully controlled; requires transformation

						induced oxidative burst in Arabidopsis [28]; dynamic changes in H_2O_2 in Arabidopsis roots induced by aluminum treatment [29]; imaging H_2O_2 accumulation in root nodules [30].	
**EPR-based detection**							
**Spin traps**							
**$^1O_2$**	DMPO	Quantitative	No	Arabidopsis	Organellar level (with extraction); limited temporal resolution due to time required for preparation and analysis.	Photoinhibition-dependent singlet oxygen and free radical production [31]	Solvation properties of spin-traps can be incompatible with biological tissue; low sensitivity requires high concentrations of spin trap (10-100 mM).
	DEPMPO	Quantitative	No	Maize		Optimization of method for apoplastic fluid extraction [32]	
	TEMPD	Quantitative	No	Pea plants		Assessing the photobleaching of chlorophyll [33].	
**•OH**	4-POBN	Qualitative and quantitative	No	Pea plants, Arabidopsis, Cucumber, Maize seedling		Diffusion of hydrogen peroxide through the chloroplast envelope [34]; hydroxyl radical production in cucumber roots and Arabidopsis seedlings [35]; ROS role in maize root wall loosening and elongation [36].	
**Spin probes**							
**Total radical production**	5-SASL	Qualitative and quantitative	No	Pea leaves	Organellar level (with extraction), e.g. thylakoid lipid vesicles. Slow preparation.	Photobleaching of chlorophyll in Light-Harvesting Complex II [33]	Lipid soluble (can be a benefit)
**$O_2^{\bullet-}$**	PTM-TC	Qualitative and quantitative	No	Arabidopsis root	Extracellular	ROS generation in root due to leaf injury [33].	Cell impermeable
	TMT-H	Qualitative and quantitative	No	Rice	Limited temporal resolution due to time required for preparation and analysis.	Influence of ethylene on ROS levels[37]; role of ROS signaling in post-submergence recovery[38]	Lipid soluble; unspecific interaction with ROS; auto-oxidation in the presence of metal ions[39]

Supplementary Table S2. Summary of methods used to detect and quantify redox markers.

Target	Method	Quantitative/ qualitative	Examples of plant species/cell types	Application	Limitation
**Cysteine oxidative modifications (general)**	'Tag-Switch': differential alkylation and subsequent identification	Qualitative or quantitative	Arabidopsis (whole cell, chloroplast, leaves, roots, shoots), wheat seeds, tomato leaves, soybean leaves.	H_2O_2-sensitive proteome of the chloroplast [40]; dormant, non-dormant, abscisic acid- or gibberellic acid-treated seed protein extracts of wheat [41]; redox-modified proteins in response to methyl jasmonate [42]; profiling the thiol redox proteome in tomato leaves after bacterial infection [43]; H_2O_2-sensitive proteins in Arabidopsis leaves [44]; ozone-induced response in soybean [45]; H_2O_2 treatment of Arabidopsis [46].	Does not reliably differentiate CysOxPTMs, incomplete or unspecific thiol blocking, incomplete reduction, sample degradation.
**Disulfide, S-S**	Modified tag-switch method including specific reduction by thioredoxin	Qualitative	Wheat endosperm, sweet tobacco, barrelclover, barley	Understanding thioredoxin-linked metabolic processes of cereal [47] [48] ; role of cystines in pollen rejection [49] ; role of thioredoxin-linked proteins in germination [50]; identification of thioredoxin targets in barley embryo [51].	Lack of specificity as Trxs or Trx-like enzymes can reduce *S*-glutathionylation and sulfhydration.
	Trx-affinity chromatography	Qualitative	Arabidopsis, spinach, potato, pea, wheat	Identification of thioredoxin targets [525354] [48]	
**Cys-sulfenic acid, SOH**	Direct Detection: specific labelling with chemical probes (dimedone, dimedone-functionalised DCP-Bio1, DYn-2, BTD)	Qualitative and globally quantitative	Arabidopsis, barrelclover	Quantification of redox-sensitive sites [55]; identification of sulfenylated proteins in H_2O_2-stressed Arabidopsis seedlings. [5657]**;** role of sulfenylation in the development and functioning of symbiotic interactions [58].	Limited to site specific quantification of the modification
	YAP1C biosensor	Qualitative and globally quantitative	Arabidopsis	H_2O_2-dependent sulfenome characterisation[59–61]	Requires transformation
**S-glutathionylation (-SSG)**	35S-radiolabelled Cys labelling	Qualitative	Arabidopsis cells, *C.reinhardtii* green algae	Impact of external oxidative stress [62] and *in vivo* targets of S-thiolation [63]	
	Biotinylated glutathione or glutathione ethyl ester	Qualitative and quantitative	Arabidopsis cells, *C.reinhardtii* green algae	Impact of external oxidative stress [62] and *in vivo* targets of glutathionylation [64]	
	Glutaredoxin affinity trapping	Qualitative	Poplar, Arabidopsis, potato and pea	Identification of plant glutaredoxin targets [65]	

**Methionine sulfoxide**	Affinity chromatography using a methionine sulfoxide reductase (MSRB)	Qualitative	Arabidopsis	H_2O_2 treatment to validate MSRB-interacting proteins as MSRB substrates. [66]	Only identifies MSRB substrates.
	Combined fractional diagonal chromatography (COFRADIC)	Qualitative and globally quantitative	Arabidopsis	Oxidative stress induced by photorespiration impairment, high light treatment [67]	
**Tryptophan oxidation**	Detection of *N*-formylkynurenin via 2D electrophoresis coupled with LC-MS/MS	Qualitative	Potato and rice	Selectivity of tryptophan oxidation [68]	
**Protein carbonylation**	Derivatisation with 2,4-dinitrophenylhydrazine, spectroscopic/ immunogenic detection and tandem MS.	Qualitative	Rice	Identification of oxidised proteins following mild external H_2O_2 exposure [69]	
	Fluorescein-5-thiosemicarbazide (FTC)	Quantitative and qualitative	Wheat	Protein carbonylation during natural leaf senescence [70]	
**Lipid oxidation products**	Chemiluminescence	Qualitative for relative changes	Almond and almond-derived foodstuffs	Monitoring lipoxidation during almond processing [71]**.**	Non-specific
	Thiobarbituric acid-reactive substances (TBARS) assay; fluorescence microscopy.	Qualitative with calibration	Beet, red cabbage, carrot, eggplant, bell pepper, radish, spinach, tomato and Arabidopsis	Improving the TBARS assay in plant tissues containing anthocyanin and other compounds [72]; mapping (potentially toxic) malondialdehyde pools in Arabidopsis. [73]	Anthocyanins can interfere with absorbance; non-specific
	Antibody-based detection	Qualitative	Spinach	Malondialdehyde causes protein modification in heat-stressed plants [74]	
	DNP-derivatization of carbonyls coupled with LC-FTICR-MS analysis	Quantitative	Arabidopsis	Determination of source fatty acids of potentially damaging short lipid peroxides in leaves [75]	.
**Sugar oxidation products**	Extraction and LC-MS/MS	Qualitative	Arabidopsis and barley	Identification of sugars as $^{\bullet}OH$ scavengers [76]	Several extraction steps required
**Nucleic acid oxidation products** (8-hydroxy-2-deoxyguanosine (8-OHdG) in DNA)	Antibody-based immunoassays	Qualitative	Arabidopsis	Quantification of 8-OHdG levels allowed characterisation of AtOGG1 as having a role in DNA damage repair .[77]	
	HPLC	Qualitative	Currant	To assess 8OHdG as a biomarker of oxidative stress and genetic stability in cryopreservation [78]	Requires extraction

Supplementary Table S3. Summary of methods used to detect and quantify NAD(P)/H.

Target	Probe	Quantitative/qualitative	Ratiometric?	Examples of plant species/ cell types	Spatial and temporal resolution	Application	Limitations
NAD(P)H	Autofluorescence	Can be quantitative with calibration [79]	No	Any	Not compartment specific; signals often dominated by mitochondria. Temporal resolution seconds to minutes	Applied to isolated plant mitochondria to quantify bound and free NADH [80]	Cannot detect oxidised forms, NAD^+ or $NADP^+$
Free[a] NADH:NAD^+	SoNar (biosensor)	Quantitative for relative changes but still requires development of calibration for absolute quantification in plants	Yes	Arabidopsis cotyledons/ seedlings	Compartment specific (cytosol and plastids). Temporal resolution seconds to minutes	Export of excess reductant form chloroplasts in the light [81]	Requires pH correction with additional fluorescent sensor. Requires genetic transformations.
Free NADH:NAD^+	Peredox-mCherry (biosensor)	Quantitative for relative changes but still requires development of calibration for absolute quantification in plants	Yes	Arabidopsis mature leaves and seedlings	Compartment specific (cytosol).[82] Temporal resolution seconds to minutes	Immune response [82], NAD^+-transporters, stomatal development, [83] reductant export from chloroplasts in the light[84]	Requires genetic transformation.
Free NADPH	iNap (biosensor)	Quantitative for relative changes but still requires development of calibration for absolute quantification in plants	Yes	Arabidopsis cotyledons/ seedlings	Compartment specific (cytosol plastids and peroxisomes). Temporal resolution seconds to minutes	Export of excess reductant from chloroplasts in the light [81]	Requires pH correction with additional fluorescent sensor. Requires genetic transformations.

[a] Free as opposed to protein bound metabolites

Supplementary Table S4. Summary of methods used to detect and quantify ROS scavengers.

Target	Method	Quantitative/ qualitative	Examples of plant species/cell types	Degree of spatial resolution	Application	Limitation
**Ascorbate**	Spectrophotometric measurement	Quantitative	Barley and Arabidopsis	Leaves	Is ascorbate content of leaf responsible for overestimation of H_2O_2 in leaves? [85]	Risk of ascorbate oxidation in sample preparation
	HPLC	Quantitative	Range of horticultural products [86]; Arabidopsis [87].	Leaves, stems, flowers	Quantitative differentiation of ascorbate at organ and growth phase of plant.	Risk of ascorbate oxidation in sample preparation
	Histochemical labelling (silver nitrate)	Qualitative	Pumpkin.	Cells and tissues of the root apex	Localization of ascorbic acid in *C. maxima* root [88].	Requires cold and acidic conditions
	Antibody labelling	Qualitative	Arabidopsis and tobacco	Chloroplasts, mitochondria and peroxisome	Ascorbate distribution in response to light stress [89].	Primary antibody cannot distinguish between ascorbate, dehydroascorbate (DHA) and monodehydroascorbate
**Glutathione**	Ellman's reagent or DTNB method	Quantitative	Arabidopsis, soybean	Organelle level (following extraction)	Role of nuclear glutathione in cell cycling[90]; glutathione estimation in crude plant extracts [91].	DTNB also reacts with other cellular thiols
	Chromatographic (HPLC, Capillary electrophoresis, LC-MS)	Quantitative	Maize, spinach, watermelon, potato, tomato and French beans	Endosperms, scutella, roots, and shoots of maize seedlings; vascular plant extracts; soft-rot of French beans	Glutathione distribution in maize seedlings in response to Cd treatment [92]; fungal infection effect on redox markers [93].	Limited specificity and alteration of the glutathione pool during extraction
	Fluorescent dyes (Monochlorobimane and monobromobimane)	Quantitative (for relative changes)	Arabidopsis	Trichoblast (root hair cells), atrichoblasts of Arabidopsis root; nuclei and cytosol of Arabidopsis root.	Direct measurement of glutathione in epidermal cells of Arabidopsis [94]; measurement of glutathione levels in intact roots of Arabidopsis [95]	Cell toxicity, reacts with other cellular thiols, cannot enter chloroplasts
	GRX1-roGFP2	Quantitative	Arabidopsis, Tobacco	Chloroplasts, mitochondria, cytosol, ER	Quantification of glutathione redox potential during seed germination and redox stress[96,97]	Measures glutathione redox potential rather than concentration; requires transformation

Supplementary Table S5. Summary of methods used to detect and quantify O_2 in plant cells and tissues.

Method	Quantitative/qualitative	Examples of plant species/cell types	Degree of spatial and temporal resolution	Application	Limitation
Clark-type electrode	Quantitative	Arabidopsis, chickpea, potato, castor plants	10 µm spatial resolution, <1 s temporal resolution	Measurement of intrinsic O_2 in different plant tissues [98]; identification of developmentally important hypoxic niche in shoot apical meristems [99]	Invasive and physically damaging, consume O_2 so may disrupt local O_2 homeostasis. Only allow local measurement.
Luminescent (optic) probes	Quantitative	Range of seeds (e.g. pea, barley, maize, sunflower), sea grass, rice and algae	50 µm spatial resolution, <1 s temporal resolution	Role of hypoxia in seed germination[100], O_2 dynamics during submergence in rice[101], O_2 dynamics during heat stress of sea grass at low tide [102]	Invasive and physically damaging; can be light sensitive. Only allow local measurement.
Human O_2-sensing cassette coupled to luciferase	Quantitative within limits	Arabidopsis seedlings and protoplasts	Slow temporal resolution (hours).	Proof of principle of O_2-responsiveness [103]	Slow temporal resolution, limited dynamic range and O_2-sensitivity.
Hypoxia-responsive promoter element-linked UnaG-mCherry reporter	Quantitative within limits (ratiometric)	*Nicotiana benthamiana*	Slow temporal resolution (hours)	Proof of principle of O_2-responsiveness [104]	Slow temporal resolution, limited dynamic range and O_2-sensitivity.

References

1 A. J. Able, D. I. Guest and M. W. Sutherland, *Plant Physiol.*, 1998, **117**, 491–499.
2 C. F. Grellet Bournonville and J. C. Díaz-Ricci, *Phytochem. Anal.*, 2011, **22**, 268–271.
3 P. M. G. Nair and I. M. Chung, *Chemosphere*, 2014, **112**, 105–113.
4 M. Rodríguez-Serrano, M. C. Romero-Puertas, D. M. Pazmino, P. S. Testillano, M. C. Risueno, L. A. Del Río and L. M. Sandalio, *Plant Physiol.*, 2009, **150**, 229–243.
5 S. Nie, H. Yue, J. Zhou and D. Xing, *PLoS One*, 2015, **10**, e0119853.
6 C. S. Bestwick, I. R. Brown, M. H. R. Bennett and J. W. Mansfield, *Plant Cell*, 1997, **9**, 209–221.
7 Y. H. Liu, C. E. Offler and Y. L. Ruan, *Front. Plant Sci.*, 2014, **5**, 1–6.
8 A. Daudi, Z. Cheng, J. A. O'Brien, N. Mammarella, S. Khan, F. M. Ausubel and G. Paul Bolwell, *Plant Cell*, 2012, **24**, 275–287.
9 L. Saleh and C. Plieth, *Plant Methods*, 2009, **5**, 1–18.
10 L. Pogačnik and N. P. Ulrih, in *Luminescence*, John Wiley & Sons, Ltd, 2012, vol. 27, pp. 505–510.
11 Z. Kolbert, A. Petô, N. Lehotai, G. Feigl, A. Ördög and L. Erdei, *Acta Biol. Szeged.*, 2012, **56**, 37–41.
12 I. Šnyrychová, F. Ayaydin and É. Hideg, *Physiol. Plant.*, 2009, **135**, 1–18.
13 C. Ortega-Villasante, L. E. Hernández, R. Rellán-Álvarez, F. F. Del Campo, R. O. Carpena-Ruiz and L. E. Hernández, *New Phytol.*, 2007, **176**, 96–107.
14 M. Potocký, M. A. Jones, R. Bezvoda, N. Smirnoff and V. Žárský, *New Phytol.*, 2007, **174**, 742–751.
15 K. A. Wilkins, J. Bancroft, M. Bosch, J. Ings, N. Smirnoff and V. E. Franklin-Tong, *Plant Physiol.*, 2011, **156**, 404–416.
16 L. Zeng, J. Zhou, B. Li and D. Xing, *Front. Plant Sci.*, 2015, **6**, 96.
17 R. Desikan, K. Last, R. Harrett-Williams, C. Tagliavia, K. Harter, R. Hooley, J. T. Hancock and S. J. Neill, *Plant J*, 2006, **47**, 907–916.
18 V. P. Ledoux Quentin, Cutsem Pierre Van, Markó Istvan E, *Plant Signal Behav.*
19 H. Wu, R. Nißler, V. Morris, N. Herrmann, P. Hu, S.-J. Jeon, S. Kruss and J. Pablo Giraldo, *Nano Lett.*, 2021, **13**, 26.
20 R. Dong, Y. Yao, D. Li, H. Zhang, W. Li, M. Molokee, Y. Liu and B. Lei, *Sensors Actuators, B Chem.*, 2020, **321**, 128643.
21 É. Hideg, T. Kálai, K. Hideg and I. Vass, *Biochemistry*, 1998, **37**, 11405–11411.
22 É. Hideg, K. Ogawa, T. Kálai and K. Hideg, *Physiol. Plant.*, 2001, **112**, 10–14.
23 C. Flors, M. J. Fryer, J. Waring, B. Reeder, U. Bechtold, P. M. Mullineaux, S. Nonell, M. T. Wilson and N. R. Baker, *J. Exp. Bot.*, 2006, **57**, 1725–1734.
24 I. Aller, N. Rouhier and A. J. Meyer, *Front. Plant Sci.*, 2013, **4**, 506.
25 T. Jubany-Mari, L. Alegre-Batlle, K. Jiang and L. J. Feldman, *FEBS Lett.*, 2010, **584**, 889–897.
26 M. Schwarzländer, M. D. Fricker and L. J. Sweetlove, *Biochim. Biophys. Acta - Bioenerg.*, 2009, **1787**, 468–475.
27 M. Exposito-Rodriguez, P. P. Laissue, G. Yvon-Durocher, N. Smirnoff and P. M. Mullineaux, *Nat. Commun.*, 2017, **8**, 4.
28 T. Nietzel, M. Elsässer, C. Ruberti, J. Steinbeck, J. M. Ugalde, P. Fuchs, S. Wagner, L. Ostermann, A. Moseler, P. Lemke, M. D. Fricker, S. J. Müller-Schüssele, B. M. Moerschbacher, A. Costa, A. J. Meyer and M. Schwarzländer, *New Phytol.*, 2019, **221**, 1649–1664.
29 A. Hernández-Barrera, A. Velarde-Buendía, I. Zepeda, F. Sanchez, C. Quinto, R. Sánchez-Lopez, A. Y. Cheung, H. M. Wu and L. Cardenas, *Sensors (Switzerland)*, 2015, **15**, 855–867.
30 D. Marino, E. Andrio, E. G. J. Danchin, E. Oger, S. Gucciardo, A. Lambert, A. Puppo and N. Pauly, *New Phytol.*, 2011, **189**, 580–592.
31 É. Hideg, C. Spetea and I. Vass, *BBA - Bioenerg.*, 1994, **1186**, 143–152.
32 J. J. Dragišic Maksimovic, B. D. Živanović, V. M. Maksimović, M. D. Mojović, M. T. Nikolic and Ž. B. Vučinić, *Plant Sci.*, 2014, **223**, 49–58.
33 M. Lingvay, P. Akhtar, K. Sebők-Nagy, T. Páli and P. H. Lambrev, *Front. Plant Sci.*, 2020, **11**, 849.
34 M. M. Borisova, M. A. Kozuleva, N. N. Rudenko, I. A. Naydov, I. B. Klenina and B. N. Ivanov, *Biochim. Biophys. Acta - Bioenerg.*, 2012, **1817**, 1314–1321.
35 S. Renew, E. Heyno, P. Schopfer and A. Liszkay, *Plant J.*, 2005, **44**, 342–347.
36 A. Liszkay, E. Van Der Zalm and P. Schopfer, *Plant Physiol.*, 2004, **136**, 3114–3123.
37 B. Steffens, A. Steffen-Heins and M. Sauter, *Front. Plant Sci.*, 2013, **4**, 179.
38 E. Yeung, H. van Veen, D. Vashisht, A. L. S. Paiva, M. Hummel, T. Rankenberg, B. Steffens, A. Steffen-Heins, M. Sauter, M. de Vries, R. C. Schuurink, J. Bazin, J. Bailey-Serres, L. A. C. J.

Voesenek and R. Sasidharan, *Proc. Natl. Acad. Sci. U. S. A.*, 2018, **115**, E6085–E6094.
39 S. Dikalov, B. Fink, M. Skatchkov and E. Bassenge, *Free Radic. Biol. Med.*, 1999, **27**, 170–176.
40 M. Muthuramalingam, A. Matros, R. Scheibe, H.-P. Mock and K.-J. Dietz, *Front. Plant Sci.*, 2013, **4**, 54.
41 N. V Bykova, B. Hoehn, C. Rampitsch, T. Banks, J.-A. Stebbing, T. Fan and R. Knox, *Proteomics*, 2011, **11**, 865–82.
42 S. Alvarez, M. Zhu and S. Chen, *J. Proteomics*, 2009, **73**, 30–40.
43 J. Parker, N. Zhu, M. Zhu and S. Chen, *J. Vis. Exp.*, 2012, 3766.
44 H. Wang, S. Wang, Y. Lu, S. Alvarez, L. M. Hicks, X. Ge and Y. Xia, *J. Proteome Res.*, 2012, **11**, 412–424.
45 A. Galant, R. P. Koester, E. a Ainsworth, L. M. Hicks and J. M. Jez, *New Phytol.*, 2012, **194**, 220–9.
46 Y. X. Pei Liu, Huoming Zhang, Hai Wang, *Proteomics*, 2014, **14**, 750–762.
47 J. H. Wong, Y. Balmer, N. Cai, C. K. Tanaka, W. H. Vensel, W. J. Hurkman and B. B. Buchanan, *FEBS Lett.*, 2003, **547**, 151–156.
48 J. H. Wong, N. Cai, Y. Balmer, C. K. Tanaka, W. H. Vensel, W. J. Hurkman and B. B. Buchanan, *Phytochemistry*, 2004, **65**, 1629–1640.
49 J. A. Juárez-Díaz, B. McClure, S. Vázquez-Santana, A. Guevara-García, P. León-Mejía, J. Márquez-Guzmán and F. Cruz-García, *J. Biol. Chem.*, 2006, **281**, 3418–24.
50 F. Alkhalfioui, M. Renard, W. H. Vensel, J. Wong, C. K. Tanaka, W. J. Hurkman, B. B. Buchanan and F. Montrichard, *Plant Physiol.*, 2007, **144**, 1559–79.
51 P. Hägglund, J. Bunkenborg, K. Maeda, B. Svensson, and B. S. Per Hagglund, Jakob Bunkenborg, Kenji Maeda and Enzyme, *J. Proteome Res.*, 2008, **7**, 5270–5276.
52 C. H. Marchand, H. Vanacker, V. Collin, E. Issakidis-Bourguet, P. Le Maréchal and P. Decottignies, *Proteomics*, 2010, **10**, 2418–2428.
53 K. Yoshida, K. Noguchi, K. Motohashi and T. Hisabori, *Plant Cell Physiol.*, 2013, **54**, 875–892.
54 Y. Balmer, W. H. Vensel, C. K. Tanaka, W. J. Hurkman, E. Gelhaye, N. Rouhier, J. P. Jacquot, W. Manieri, P. Schürmann, M. Droux and B. B. Buchanan, *Proc. Natl. Acad. Sci. U. S. A.*, 2004, **101**, 2642–2647.
55 J. Huang, P. Willems, B. Wei, C. Tian, R. B. Ferreira, N. Bodra, S. A. Martínez Gache, K. Wahni, K. Liu, D. Vertommen, K. Gevaert, K. S. Carroll, M. Van Montagu, J. Yang, F. Van Breusegem and J. Messens, *Proc. Natl. Acad. Sci. U. S. A.*, 2019, **116**, 20256–20261.
56 S. Akter, J. Huang, N. Bodra, B. De Smet, K. Wahni, D. Rombaut, J. Pauwels, K. Gevaert, K. Carroll, F. Van Breusegem and J. Messens, *Mol. Cell. Proteomics*, 2015, **14**, 1183–1200.
57 S. Akter, S. Carpentier, F. Van Breusegem and J. Messens, *Biochem. Biophys. Reports*, 2017, **9**, 106–113.
58 E. Oger, D. Marino, J. M. Guigonis, N. Pauly and A. Puppo, *J. Proteomics*, 2012, **75**, 4102–4113.
59 C. Waszczak, S. Akter, D. Eeckhout, G. Persiau, K. Wahni, N. Bodra, I. Van Molle, B. De Smet, D. Vertommen, K. Gevaert, G. De Jaeger, M. Van Montagu, J. Messens and F. Van Breusegem, *Proc Natl Acad Sci U S A*, 2014, **111**, 11545–11550.
60 B. Wei, P. Willems, J. Huang, C. Tian, J. Yang, J. Messens and F. Van Breusegem, *Front. Plant Sci.*, 2020, **11**, 777.
61 B. De Smet, P. Willems, A. D. Fernandez-Fernandez, S. Alseekh, A. R. Fernie, J. Messens and F. Van Breusegem, *Plant J.*, 2019, **97**, 765–778.
62 D. P. D. Dixon, M. Skipsey, N. M. N. Grundy, R. Edwards, B. Sciences and U. Kingdom, *Plant Physiol.*, 2005, **138**, 2233–2244.
63 L. Michelet, M. Zaffagnini, H. Vanacker, P. Le Maréchal, C. Marchand, M. Schroda, S. D. Lemaire and P. Decottignies, *J. Biol. Chem.*, 2008, **283**, 21571–21578.
64 M. Zaffagnini, M. Bedhomme, H. Groni, C. H. Marchand, C. Puppo, B. Gontero, C. Cassier-Chauvat, P. Decottignies and S. D. Lemaire, *Mol. Cell. Proteomics*, 2012, **11**, M111.014142.
65 N. Rouhier, A. Villarejo, M. Srivastava, E. Gelhaye, O. Keech, M. Droux, I. Finkemeier, G. Samuelsson, K. J. Dietz, J.-P. Jacquot and G. Wingsle, *Antioxid. Redox Signal.*, 2005, **7**, 919–29.
66 L. Tarrago, S. Kieffer-Jaquinod, T. Lamant, M. Marcellin, J. Garin, N. Rouhier and P. Rey, *Antioxidants Redox Signal.*, 2012, **16**, 79–84.
67 S. Jacques, B. Ghesquière, P. J. De Bock, H. Demol, K. Wahni, P. Willems, J. Messens, F. Van Breusegem and K. Gevaert, *Mol. Cell. Proteomics*, 2015, **14**, 1217–1229.
68 I. M. Møller and B. K. Kristensen, *Free Radic. Biol. Med.*, 2006, **40**, 430–435.
69 B. K. Kristensen, P. Askerlund, N. V. Bykova, H. Egsgaard and I. M. Møller, *Phytochemistry*, 2004, **65**, 1839–1851.
70 M. Havé, L. Leitao, M. Bagard, J. F. Castell and A. Repellin, *Plant Biol.*, 2015, **17**, 973–979.
71 S. Tazi, A. Puigserver and E. H. Ajandouz, *Food Chem.*, 2009, **116**, 999–1004.
72 D. M. Hodges, J. M. DeLong, C. F. Forney and R. K. Prange, *Planta*, 1999, **207**, 604–611.
73 L. Mène-Saffrané, C. Davoine, S. Stolz, P. Majcherczyk and E. E. Farmer, *J. Biol. Chem.*, 2007, **282**, 35749–35756.
74 Y. Yamauchi, A. Furutera, K. Seki, Y. Toyoda, K. Tanaka and Y. Sugimoto, *Plant Physiol. Biochem.*, 2008, **46**, 786–793.
75 J. Mano, S. Khorobrykh, K. Matsui, Y. Iijima, N. Sakurai, H. Suzuki and D. Shibata, *Plant Biotechnol.*, 2014, **31**, 535–543.

76 A. Matros, D. Peshev, M. Peukert, H.-P. Mock and W. Van den Ende, *Plant J.*, 2015, **82**, 822–839.
77 H. Chen, P. Chu, Yuliang Zhou, Y. Li, J. Liu, Y. Ding, E. W. T. Tsang, L. Jiang, K. Wu and S. Huang, *J. Exp. Bot.*, 2012, **63**, 4107–4121.
78 J. W. Johnston, I. Pimbley, K. Harding and E. E. Benson, *Cryo-Letters*, 2010, **31**, 1–13.
79 N. Ma, M. A. Digman, L. Malacrida and E. Gratton, *Biomed. Opt. Express*, 2016, **7**, 2441.
80 M. R. Kasimova, *Plant Cell Online*, 2006, **18**, 688–698.
81 S. Lim, C. P. Voon and B. L. Lim, *Nat. Commun.*, 2020, 3238.
82 J. Steinbeck, P. Fuchs, Y. L. Negroni, M. Elsässer, S. Lichtenauer, Y. Stockdreher, E. Feitosa-Araujo, J. B. Kroll, J. O. Niemeier, C. Humberg, E. N. Smith, M. Mai, A. Nunes-Nesi, A. J. Meyer, M. Zottini, B. Morgan, S. Wagner and M. Schwarzländer, *Plant Cell*, 2020, **32**, 3324–3345.
83 E. Feitosa-Araujo, P. da Fonseca-Pereira, M. M. Pena, D. B. Medeiros, L. Perez de Souza, T. Yoshida, A. P. Weber, W. L. Araújo, A. R. Fernie, M. Schwarzländer and A. Nunes-Nesi, *Plant J.*, 2020, **104**, 1149–1168.
84 M. Elsässer, E. Feitosa-Araujo, S. Lichtenauer, S. Wagner, P. Fuchs, J. Giese, F. Kotnik, M. Hippler, A. J. Meyer, V. G. Maurino, I. Finkemeier, M. Schallenberg-Rüdinger and M. Schwarzländer, *bioRxiv*, 2020, 1–45.
85 S. Veljovic-Jovanovic, G. Noctor and C. H. Foyer, *Plant Physiol. Biochem.*, 2002, **40**, 501–507.
86 V. Spínola, B. Mendes, J. S. Câmara and P. C. Castilho, *Anal. Bioanal. Chem.*, 2012, **403**, 1049–1058.
87 N. Kka, J. Rookes and D. Cahill, *Plant Growth Regul.*, 2017, **81**, 283–292.
88 R. Liso, M. C. De Tullio, S. Ciraci, R. Balestrini, N. La Rocca, L. Bruno, A. Chiappetta, M. B. Bitonti, P. Bonfante and O. Arrigoni, *J. Exp. Bot.*, 2004, **55**, 2589–2597.
89 B. Zechmann, M. Stumpe and F. Mauch, *Planta*, 2011, **233**, 1–12.
90 T. K. Pellny, V. Locato, P. D. Vivancos, J. Markovic, L. De Gara, F. V. Pallardó and C. H. Foyer, *Mol. Plant*, 2009, **2**, 442–456.
91 I. K. Smith, T. L. Vierheller and C. A. Thorne, *Anal. Biochem.*, 1988, **175**, 408–413.
92 W. E. Rauser, R. Schupp and H. Rennenberg, *Plant Physiol.*, 1991, **97**, 128–138.
93 I. Muckenschnabel, B. Williamson, B. A. Goodman, G. D. Lyon, D. Stewart and N. Deighton, *Planta*, 2001, **212**, 376–381.
94 A. J. Meyer and M. D. Fricker, *J. Microsc.*, 2000, **198**, 174–181.
95 M. D. Fricker, M. May, A. J. Meyer, N. Sheard and N. S. White, *J. Microsc.*, 2000, **198**, 162–173.
96 T. Nietzel, J. Mostertz, C. Ruberti, G. Nee, P. Fuchs, S. Wagner, A. Moseler, S. Muller-Schussele, A. Benamar, G. Poschet, M. Buttner, I. M. Møller, C. H. Lillig, D. Macherel, M. Wirtz, R. Hell, I. Finkemeier, A. J. Meyer, F. Hochgräfe and M. Schwarzländer, *Proc Natl Acad Sci U S A*, 2020, **117**, 741–751.
97 L. Marty, W. Siala, M. Schwarzlä Nder C, M. D. Fricker, M. Wirtz, L. J. Sweetlove, Y. Meyer, A. J. Meyer, J.-P. Reichheld and R. Diger Hell, *Proc Natl Acad Sci U S A*, 2009, **106**, 9109–9114.
98 D. A. Weits, J. T. van Dongen and F. Licausi, *New Phytol.*, 2021, **229**, 24–35.
99 D. A. Weits, A. B. Kunkowska, N. C. W. Kamps, K. M. S. Portz, N. K. Packbier, Z. Nemec Venza, C. Gaillochet, J. U. Lohmann, O. Pedersen, J. T. van Dongen and F. Licausi, *Nature*, 2019, **569**, 714–717.
100 H. Rolletschek, A. Stangelmayer and L. Borisjuk, *Sensors*, 2009, **9**, 3218–3227.
101 Y. Mori, Y. Kurokawa, M. Koike, A. I. Malik, T. D. Colmer, M. Ashikari, O. Pedersen and K. Nagai, *Plant Cell Physiol.*, 2019, **60**, 973–985.
102 O. Pedersen, T. D. Colmer, J. Borum, A. Zavala-Perez and G. A. Kendrick, *New Phytol.*, 2016, **210**, 1207–1218.
103 S. Iacopino, S. Jurinovich, L. Cupellini, L. Piccinini, F. Cardarelli, P. Perata, B. Mennucci, B. Giuntoli and F. Licausi, *Plant Physiol.*, 2019, **179**, 986–1000.
104 G. Panicucci, S. Iacopino, E. De Meo, P. Perata and D. A. Weits, *Biosensors*, 2020, **10**, 197.
